# Monitoring the hyporheic zone: a global review and strategic directions for improvement

**DOI:** 10.1007/s10661-026-15291-4

**Published:** 2026-04-21

**Authors:** Daniel da Silva Andrade, Tiziana Di Lorenzo, Silvia Vendruscolo Milesi, Luiz Ubiratan Hepp, Riccardo Mugnai

**Affiliations:** 1 Programa de Pós-Graduação em Ciências Ambientais, Universidade Federal do Maranhão (UFMA), BR 222 Km 4, S/N, Bairro Boa Vista, 65500-000 Chapadinha, MA Brazil; 2 Laboratório de Organismos Aquáticos, Curso Oceanografia, Centro de Ciências Biológicas e da Saúde (CCBS), Universidade Federal do Maranhão (UFMA), Av. Dos Portugueses 1966, Cidade Universitária Dom Delgado, 65080-805 São Luís, MA Brazil; 3 Research Institute On Terrestrial Ecosystems of the National Research Council of Italy (IRET CNR), 50019 Florence, Italy; 4 National Biodiversity Future Center (NBFC), 90133 Palermo, Italy; 5 Emil Racovi¸tˇa” Institute of Speleology, 400535 Cluj-Napoca, Romania; 6 Centre for Ecology, Evolution and Environmental Changes (CE3C), CHANGE—Global Change and Sustainability Institute, Departamento de Biologia, Faculdade de Ciências, Universidade de Lisboa, Campo Grande, 1749-016 Lisbon, Portugal; 7 Laboratório de Indicadores Ambientais, Universidade Federal de Mato Grosso do Sul (UFMS), Campus Três Lagoas, Av. Ranulpho Marques Leal, 3484, 79613-000 Três Lagoas, MS Brazil; 8 Programa de Pós-Graduação em Biodiversidade e Conservação, Cidade Universitária Dom Delgado, Universidade Federal do Maranhão (UFMA), Av. Dos Portugueses 1966, 65080-805 São Luís, MA Brazil; 9 Laboratório de Plâncton, Departamento de Hidrobiologia, Universidade Federal de São Carlos (UFSCar), Rodovia Washington Luís, Km 235, 13565-905 São Carlos, SP Brazil; 10 Present Address: Programa de Pós-Graduação em Ciências Biológicas (Zoologia), Instituto de Biociências, Universidade Estadual Paulista “Júlio de Mesquita Filho” (UNESP), R. Prof. Dr. Antônio Celso Wagner Zanin, 250, Distrito de Rubião Junior, 18618-689 Botucatu, SP Brazil; 11 Programa de Pós-Graduação em Biodiversidade e Meio Ambiente, Universidade Federal da Grande Dourados (UFGD), R. João Rosa Góes- 1761 - Vila Progresso, 79825-070 Dourados, MS Brazil

**Keywords:** Biomonitoring, Groundwater, Interstitial environment, Environmental legislation, Environmental impact, Hyporheic exchange

## Abstract

The hyporheic zone (HZ) is a crucial interface in riverbeds that links surface and subterranean waters, supporting a unique ecological community. While recent reviews have explored theoretical and ecological aspects of HZ, this study focuses on evaluating the advances in monitoring the quality and conservation status. We conducted a global systematic review, screening 16,135 articles and retaining a final data pool of 102 peer-reviewed studies published between 1989 and 2024. We address four main areas: (i) spatial and temporal distribution, (ii) monitoring activities, (iii) methodologies employed, and (iv) the impact of legislation. Despite advances in technical knowledge, HZ monitoring efforts remain quantitatively limited. Geographically, research is highly skewed, with Europe (51.0%), Asia (21.6%), and North America (19.6%) dominating the field, while other regions are severely underrepresented. Taxonomically, studies predominantly focus on the entire invertebrate community (35.5%) or microorganisms (30.3%), whereas efficient trait-based approaches remain scarce (6.6%). Methodologically, sampling is largely confined to shallow depths (up to − 50 cm). Furthermore, the enactment of environmental legislation showed a localized, rather than global, stimulus on monitoring outputs. This study emphasizes the urgent need for standardized methodologies, integrated surface–groundwater approaches, and targeted efforts to ensure effective water resource management. Finally, we propose a strategic roadmap with short-, medium-, and long-term actions to guide these improvements.

**Supplementary Information:**

The online version contains supplementary material available at 10.1007/s10661-026-15291-4.

## Introduction

Freshwater security is one of the most pressing global challenges of the twenty-first century (Vörösmarty et al., 2010). Rapid population growth, urbanization, agricultural intensification, and climate change are placing unprecedented pressure on rivers, lakes, wetlands, and groundwater worldwide (Moghaddam et al., 2025; Steffen et al., 2015). Global water demand is projected to increase substantially in the coming decades, exacerbating water scarcity and amplifying conflicts between human consumption, food production, energy generation, and ecosystem integrity (e.g., Boretti & Rosa, 2019). These pressures directly threaten biodiversity, ecosystem functioning, and the provision of essential ecosystem services, including drinking water supply and nutrient cycling (e.g., Saccò et al., 2024). The management of water resources, public health interventions, and measures to mitigate the environmental impact of uncontrolled population growth remain critical areas of study (Borsetto et al., 2025).

Water resource management has progressively shifted from approaches focused solely on quantitative allocation and physicochemical compliance toward more integrated frameworks that incorporate ecological perspectives (Birk et al., 2020; Dykes et al., 2025; Mohammadpour et al., 2025). Biomonitoring has become a central component of contemporary freshwater assessment frameworks worldwide. It is especially valuable because it integrates the cumulative responses of biological communities over time, whereas traditional monitoring provides only short-term snapshots that may fail to detect cumulative impacts. However, an important exception remains regarding groundwater (Di Lorenzo et al., 2024). In Europe, groundwater assessment under the Water Framework Directive and the Groundwater Directive is still largely focused on quantitative and chemical status, with no formal integration of biological quality elements, despite increasing scientific evidence supporting their relevance (Di Lorenzo et al., 2024). In contrast, other countries, notably Australia, have explicitly incorporated groundwater-dependent ecosystems and subterranean biota into their monitoring and management frameworks (Environmental Protection Authority, 2016).

However, in recent decades, there has been a growing interest in subsurface waters, especially the hyporheic zone (HZ) (Mugnai et al., 2015a). The term was originally proposed by Orghidan (1959, 2010), who described it as a discrete interfacial compartment in the riverbed that hosts a unique community, functioning as a critical part of river ecosystems by connecting surface water and groundwater. Later, Boulton et al. (1998) defined it as an ecotone, or transition zone, between surface water and groundwater. The HZ is a dynamic transition boundary where the bidirectional exchange between surface water and a fixed sediment bed drives small-scale vertical fluxes of water and solutes, distinguishing it from both surface flow and regional groundwater pathways (Tonina & Buffington, 2007). Recent fundamental studies have emphasized how bedform morphology, sediment characteristics, and pressure-driven “pumping” processes regulate hyporheic exchange, solute residence times, and microbial activity. These studies highlight the strong feedbacks between hydrodynamics, biofilm development, and biogeochemical cycling that are central to river functioning and restoration (e.g., Cook et al., 2020). In addition to the HZ in lotic environments, a similar ecotone, the hypolentic zone, exists in lentic waters, serving as the interface between surface water and groundwater in those systems (Winter, 2001).

From a biological perspective, the HZ serves as a nursery and refuge for benthic species, a feeding area, and a habitat for species exclusive to this environment (Datry & Larned, 2008; Stubbington et al., 2009). The hyporheic community, referred to as the hyporheos, represents a unique entity that is distinct from those in surface waters and groundwater, although it shares characteristics with both ecosystems (Peralta-Maraver et al., 2018). Within the HZ, stygobites (species that complete their life cycle in subterranean aquatic habitats) are commonly observed in upwelling areas, while surface taxa are typically found in downwelling zones (Williams et al., 2010).

The hydrological, chemical, biological, and metabolic characteristics of the HZ are specific to this zone and distinct from both surface waters and groundwater (Krause et al., 2011; Lewandowski et al., 2019; Tonina & Buffington, 2023). In general, the HZ represents an area of significant importance for productivity, nutrient cycling (e.g., Boulton et al., 1998; Brunke & Gonser, 1997; Findlay, 1995), and pollutant processing (Lewandowski et al., 2019), housing highly diverse invertebrate communities (Boulton et al., 2004; Gibert, 1991). Furthermore, in the last decade, the HZ has gained attention for its role in public health; it is an area prone to the accumulation of toxic substances and can act as a reservoir and transport pathway for zoonotic agents and viruses, such as COVID-19 (Kumar et al., 2020; Mugnai et al., 2015a, b).

The HZ is subject to numerous impacts, whether physical, chemical, or microbiological (Boulton et al., 1998; Hancock, 2002; Mugnai et al., 2015b). Physical impacts, such as sediment clogging resulting from the deposition of particles in stream and riverbeds, can significantly reduce the permeability of sediments in interstitial environments, disrupting hyporheic microbial processes and the circulation of nutrients (Nogaro et al., 2010; Schälchli, 1992). Recent advances highlight how changes in sediment structure and flow dynamics strongly regulate contaminant transport and attenuation in subsurface exchange zones, emphasizing the role of residence time distributions and reactive interfaces in controlling pollutant fate (e.g., Saeibehrouzi et al., 2025). Chemical pressures include heavy metals, pharmaceutical residues, and contaminants derived from urban, agricultural, and industrial activities, which can accumulate within hyporheic sediments and alter microbial community composition and ecosystem functioning (Redžović et al., 2023; Rutere et al., 2020; Sonne et al., 2018). The occurrence and transport of microplastics within the HZ have recently emerged as a key research focus (Stride et al., 2025). Regarding microbial pollution, the presence of pathogenic bacteria such as *Escherichia coli* and *Salmonella* is of significant concern, as this contamination poses a serious risk to public health and requires urgent mitigation measures (Mugnai et al., 2015b).

Despite the ecological importance of the HZ for the sustainability of water resources and public health, and the substantial volume of theoretical research published over the past 70 years, specific legislation for its protection exists in only a few regions. These include the USA (Safe Drinking Water Act Amendments of 1996), the European Union (Groundwater Directive 2006/118/EC), and Australia (Water Act 2007). Furthermore, there is a lack of comprehensive information regarding the current status of HZ monitoring and the most commonly utilized methodologies (Malard et al., 2002). Addressing this knowledge gap is essential for standardizing monitoring activities globally and developing effective policies to mitigate impacts on the HZ.

Previous bibliographical reviews have primarily focused on the theoretical and ecological aspects of the HZ (e.g., Boulton et al., 1998; Mugnai et al., 2015a; Pascuale et al., 2024). While this foundational knowledge is essential, there remains a clear gap regarding the practical implementation of monitoring strategies to assess ecological status, evaluate degradation, and enforce conservation measures. To address this gap, this study provides a systematic review of the scientific literature on HZ monitoring at a global scale. Specifically, we aim to (i) analyze the spatial and temporal distribution of monitoring efforts, (ii) identify the types of monitoring activities conducted, (iii) examine the methodologies employed, and (iv) evaluate the extent to which legislative frameworks influence monitoring practices. Ultimately, this work seeks to establish a foundational knowledge base and identify critical data gaps, informing the development of environmental policies necessary for the sustainable management of hyporheic zones. Furthermore, we propose actionable strategies that can be implemented in the short, medium, and long term to support this process.

## Materials and methods

### Literature search and eligibility criteria

**Fig. 1:**
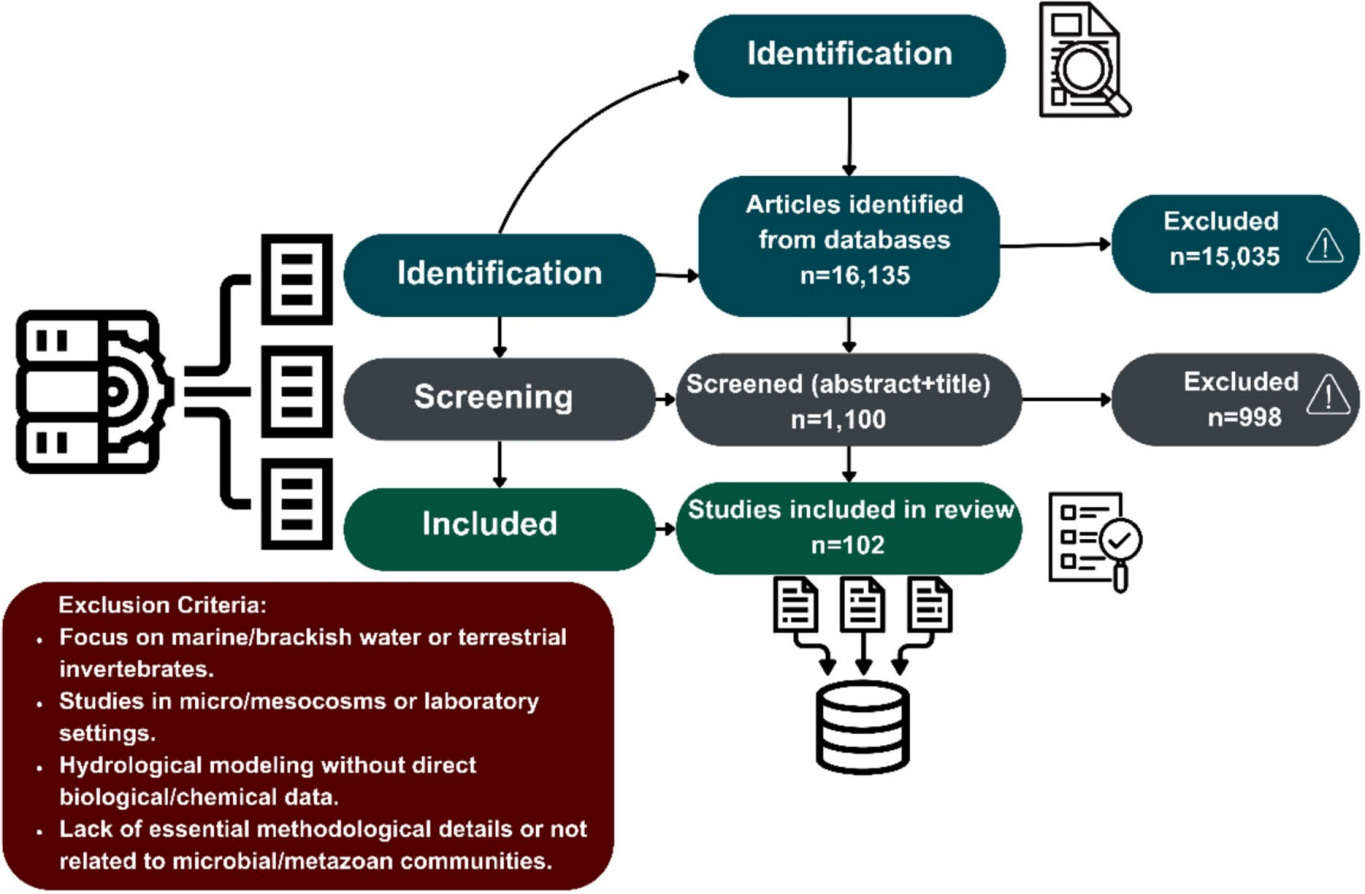
Preferred Reporting Items for Systematic Reviews and Meta-Analyses (PRISMA) for this study. Flow diagram depicting the flow of information through the different phases of the systematic literature survey

To conduct a systematic and comprehensive literature search, we followed the Preferred Reporting Items for Systematic Reviews and Meta-Analyses (PRISMA) guidelines (Page et al., 2021) (Fig. 1). First, we developed a literature search protocol to identify peer-reviewed articles in indexed journals. Initially, we compiled a core set of relevant studies to refine the final search string. This core was obtained through a combination of backward citation tracking from key papers independently selected by the authors and an independent literature search conducted by the first author using Google Scholar. After reviewing the initially extracted papers, we reached a consensus among co-authors on the final search string, which was then applied across multiple scholarly databases, including Web of Science, PubMed, Google Scholar, and Scopus: TS = (“Hyporheic” AND “Groundwater” AND “Interstitial environment”) AND TS = (“Biomonitoring” AND “Monitoring”).

We limited our search to articles, review papers, and book chapters written in English. No chronological restrictions were applied to the search, and all relevant publications up to 2024 were included. Thus, the time period analyzed strictly reflects the natural availability of published literature on this topic. We initially extracted 16,135 papers. This high initial number was driven by the comprehensive nature of the databases consulted (e.g., Google Scholar) and the interdisciplinary overlap of our search terms with marine, soil, and purely hydrogeological literature. We manually checked for duplicates and thoroughly screened all titles and abstracts. Rigorous application of the exclusion criteria was necessary to filter out this non-target “noise” and isolate true in situ freshwater studies, excluding clearly irrelevant references based on the following criteria: (1) studies focusing on marine, brackish water taxa, or non-aquatic (soil) invertebrates; (2) research conducted in micro- or mesocosms or under laboratory conditions; (3) studies centered on hydrogeological modeling lacking direct biological and chemical measurements; and (4) documents lacking critical methodological details or unrelated to microbial or metazoan communities.

### Data categorization

The categories include (1) general data (title, authors, year of publication, and journal) to document the bibliometric aspects of HZ monitoring research; (2) broad geographic region (North America, Central and South America, Europe (European Union), Oceania (Australia), Asia, and Africa) to assess global research distribution and identify underrepresented regions; (3) coordinates of the sampling sites to enable spatial analysis and heatmap visualization of monitoring efforts; (4) location of the study to provide a finer-scale understanding of geographic distribution; (5) timing of the paper’s publication in relation to the enactment of key laws (before and after the Groundwater Directive 2006/118/EC in Europe, the Safe Drinking Water Act Amendments of 1996 in the USA, and the Water Act 2007 in Australia) to evaluate the potential influence of environmental legislation on HZ research trends; (6) investigated group (crustaceans, insects, entire invertebrate community, and other taxa, including microorganisms and non-arthropod invertebrates) to analyze taxonomic focus and the use of trait-based approaches in monitoring. To prevent data overlapping, these categories were mutually exclusive. Studies focusing solely on a specific taxon were assigned only to that group, whereas studies evaluating broader benthic assemblages were categorized exclusively as the “entire invertebrate community; (7) ecological classification of invertebrate fauna (epigean, hypogean, hyporheic, and phreatic) to explore the distribution of studied taxa across different aquatic habitats; (8) sampling depth (0 to − 10, − 10 to − 20, − 20 to − 30, − 30 to − 40, − 40 to − 50, and >  − 50 cm) to assess how studies explore different vertical strata of the HZ and identify potential biases toward specific depths; (9) investigated impacts (heavy metals, industrial pollution, antibiotics, urban pollution, microplastics, agricultural pollution, clogging, eutrophication, viruses, bacteria, salinity, and others) to determine the primary environmental concerns addressed in HZ research and identify gaps in impact assessment; (10) water body recovery to track studies focusing on HZ restoration and post-disturbance monitoring; (11) correlation with surface water (whether the study also considers characteristics of surface waters) to evaluate how frequently HZ research integrates surface–subsurface interactions; (12) sampling techniques employed (permanent station, mini-piezometer, Bou-Rouch, and others, such as cores and traps) to document methodological approaches and assess the evolution of sampling strategies over time; (13) sampling effort (number of sampling sites and number of samples) to compare the scope of different studies and assess their representativeness; and (14) sampling over time (whether temporal sampling replication exists) to differentiate between snapshot studies and long-term monitoring efforts.

This information was organized in an Excel file as a single dataset (Online Resource 1). These classes were deliberately organized to address the four key analytical dimensions of this review: (i) the spatial and temporal distribution of monitoring efforts globally, (ii) monitoring activities, (iii) methodologies employed, and (iv) the impact of legislation on monitoring activities.

### Data analysis

Bibliometric variables were quantitatively analyzed to characterize the structure of the compiled dataset. Publication year, journal, geographic region, and study type were treated as either categorical or continuous variables and were summarized using frequency counts, proportions, and temporal distributions. Publication clustering and regional representation were assessed by calculating the relative contribution (%) of each category to the total dataset. Temporal publication trends were evaluated using exponential regression models, while differences among categorical variables were tested using chi-square analyses.

To evaluate publication trends in detail, the data were categorized by the year and type (biological versus abiological) of publication. We then analyzed the temporal distribution of publications using exponential growth models, as a preliminary visual inspection suggested a non-linear increase over time. Model selection was based on a visual assessment of fit and goodness-of-fit metrics. Separate models were applied to both the annual and cumulative numbers of publications. The percentages for each category were calculated based on the total number of studies and visualized graphically. To determine whether the observed differences among biological groups, ecological classifications, environmental impacts, and sampling methods were statistically significant, chi-square tests were applied.

Additionally, to evaluate whether legislative enactments influenced research output on HZ monitoring, we conducted chi-square goodness-of-fit tests. Studies were categorized based on their publication timing relative to key legislative milestones: the Safe Drinking Water Act Amendments of 1996 in the USA (U.S. Environmental Protection Agency, 1997), the Groundwater Directive 2006/118/EC in the European Union (European Community, 2006), and the Water Act 2007 in Australia (Water Act., 2007). For each region, we compiled the number of studies published before and after the respective legislation and performed separate chi-square tests to assess the statistical significance of the observed differences. These tests evaluated whether the distribution of studies before and after the legislation deviated from an expected uniform distribution, allowing us to infer potential policy-driven changes in research output. All statistical analyses, model fitting, and graphical outputs were performed in R version 4.2.3 (R Core Team, 2023).

The geographic distribution of publications was visualized through a heatmap generated in QGIS version 3.34.3, using the geographic coordinates of the sampling sites. Heatmap parameters, such as the color scale and the search radius (radius of influence), were subsequently adjusted to accurately represent the spatial density of the studies.

## Results

### Spatial and temporal distribution of monitoring efforts globally

#### Publication trends

An analysis of the literature on HZ environmental quality monitoring revealed that the first relevant study was published in 1989 (Ward et al., 1989), followed by a second in 1996 (Lafont et al., 1996). Overall, the analyzed period spans from 1989 to December 2024, encompassing a total of 102 papers.

These studies were published across 68 different journals. Notably, 25.5% of the total articles were concentrated in just four journals: Science of the Total Environment (*n* = 10), Hydrobiologia (*n* = 8), Applied and Environmental Microbiology (*n* = 4), and Ecological Indicators (*n* = 4). The remaining articles were widely distributed across 64 additional journals; a distribution pattern we analyzed to evaluate research fragmentation in the field.

**Fig. 2:**
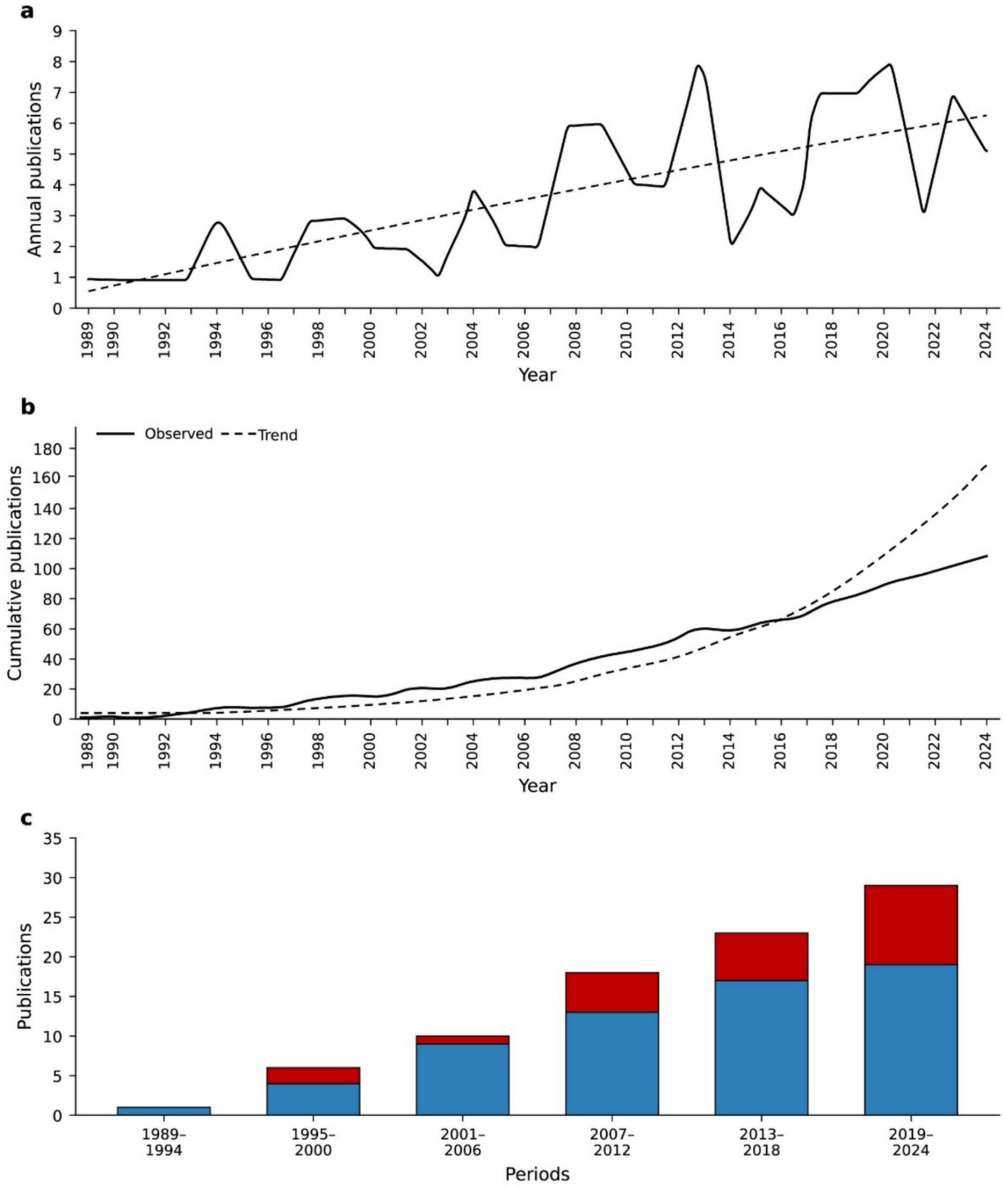
Temporal trend of publications from 1989 to 2024. **a** Annual number of publications, with the dashed line indicating the exponential growth trend. **b** Cumulative number of publications, with the dashed line representing the overall exponential increase over the period. **c** Temporal distribution of studies focused on hyporheic zone monitoring, categorized by data type: blue bars represent biological monitoring, while red bars indicate abiological data

The number of publications exhibited a clear upward trajectory over time. An exponential growth model best described this annual increase (number of publications = 1.03e^0.066*year^; *R*^2^ = 0.60; Fig. 2a). Similarly, a strong exponential increase was the most suitable model for the cumulative number of publications (cumulative publications = 2.87e^0.139*year^; *R*^2^ = 0.95; Fig. 2b).

Both biomonitoring and environmental monitoring studies have increased over time; however, the number of environmental monitoring publications has risen more prominently since 2007 (Fig. 2c).

#### Geographic distribution of studies

**Fig. 3:**
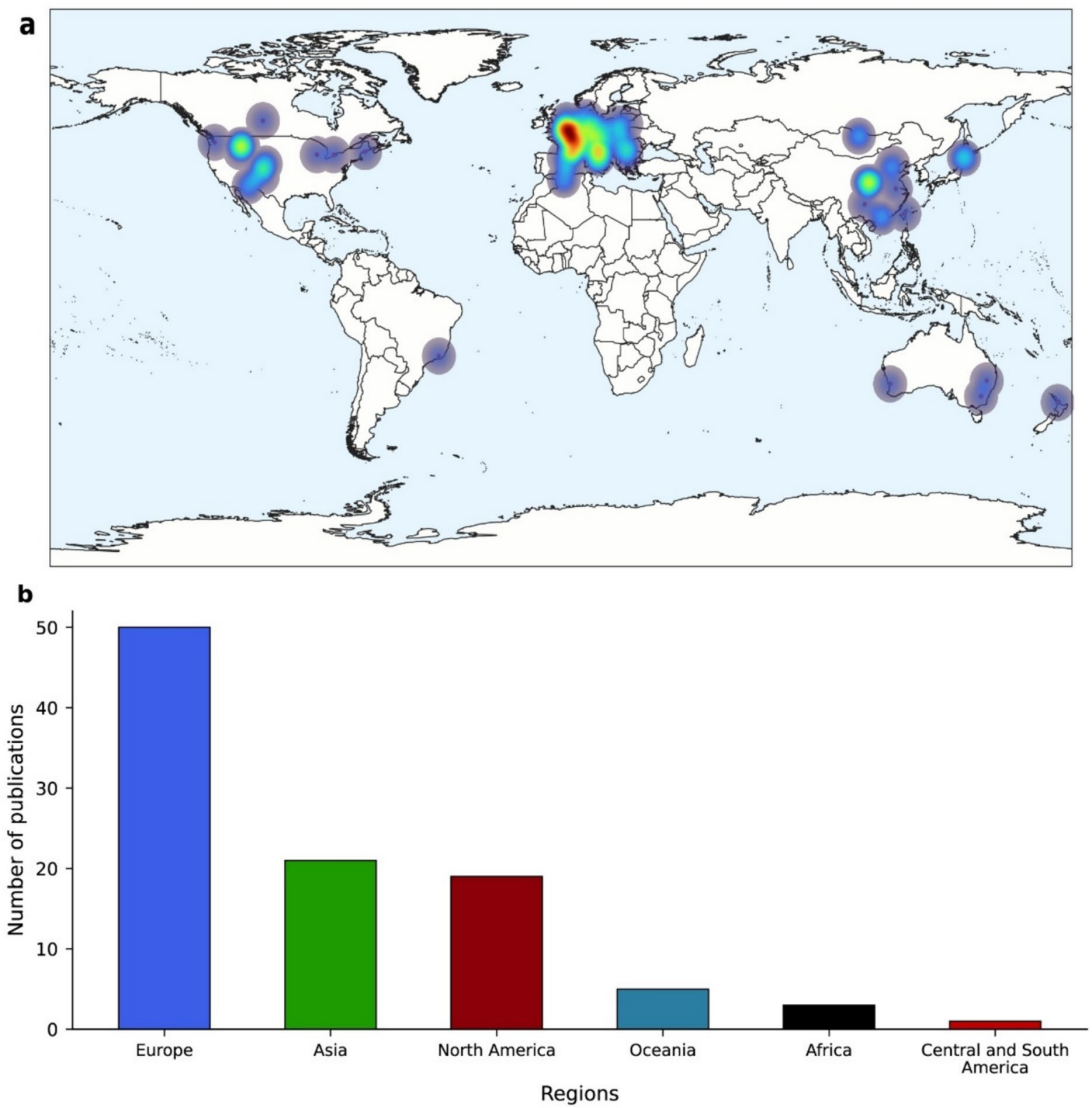
Global distribution and regional breakdown of research efforts. **a** Heatmap overlaid on a world map illustrating the spatial concentration of studies (red = high research effort; blue = low research effort). **b** Number of publications per geographical region

The geographic distribution analysis revealed that Europe accounted for more than half of the studies (51.0%), followed by Asia (21.6%, primarily represented by China), and North America (19.6%). Other regions were notably less represented: Oceania (4.9%), Africa (2.0%), and Central and South America (1.0%) (Fig. 3a, b). Publications from Asia were the most recent; the first study from this region, which focused on the impact of suspended sediments on HZ functions in the Kharaa River catchment (Mongolia), was published in 2012.

### Monitoring activities

#### Investigated categories

**Fig. 4:**
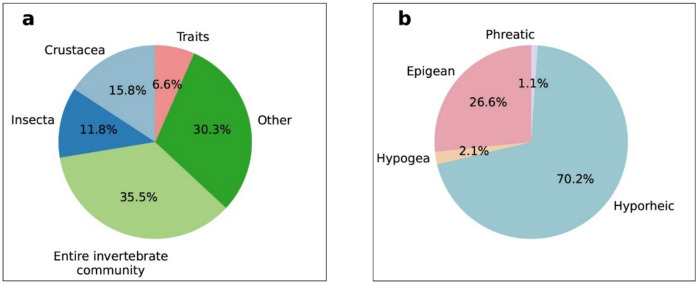
Overview of the taxonomic and habitat categories from the reviewed studies. **a** Percentage of taxa investigated in the studies included in the review. **b** Percentage of fauna by habitat type

Regarding biological assessments, HZ monitoring studies have predominantly focused on broader biological communities rather than specific taxa. The most frequently investigated groups were the entire invertebrate community and a broader category of “other taxa” (which encompasses microorganisms such as bacteria and viruses, as well as non-arthropod invertebrates like annelids), representing 35.5% and 30.3% of the studies, respectively (*χ*^2^ = 30.5, *p* < 0.001). In contrast, studies specifically targeting crustaceans and insects accounted for only 27.6% of the total. Although trait-based analyses are widely recommended to overcome taxonomic limitations and improve the efficiency of ecological assessments, these approaches were notably underrepresented in our dataset, appearing in only 6.6% of the publications (Fig. 4a).

#### Ecological classification of fauna

Regarding ecological classification, most studies (70.0%) focused on hyporheic fauna as defined by Orghidan (1959), indicating a statistically significant preference (*χ*^2^ = 124.3, *p* < 0.001). Only a small proportion of the literature included phreatic species (1.1%) or employed the broader term “hypogean” (2.1%) (Fig. 4b). Additionally, 26.6% of the publications integrated data from both surface and subsurface systems by combining epigean and hyporheic fauna, which provided a more holistic understanding of the aquatic ecosystem.

#### Environmental impacts investigated

**Fig. 5:**
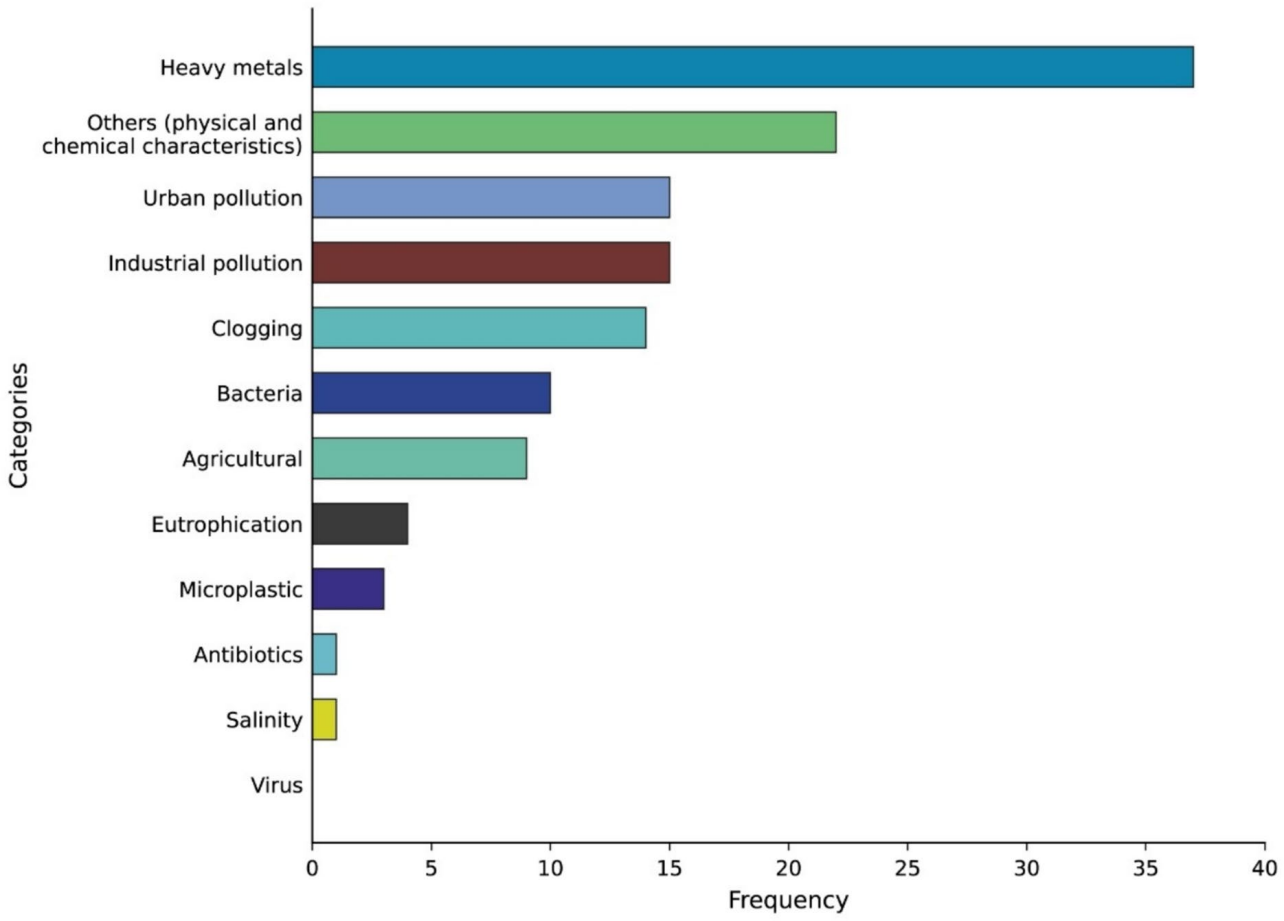
Distribution of research focus on environmental impacts. The horizontal bar chart illustrates the number of publications investigating different categories of environmental impacts

Among the investigated impacts, heavy metal contamination was the most frequently addressed concern in hyporheic environments (37.2%). This predominance showed a statistically significant difference across categories (*χ*^2^ = 91.5, *p* < 0.001; Fig. 5). The second most common category was “other” (22.5%), which encompassed studies on the physicochemical characteristics of hyporheic water, such as oxygen and nitrogen concentrations. Industrial and urban pollution were each examined in 14.7% of the studies. These were followed by clogging (13.7%) and microbes (9.8%). Agriculture-related impacts (8.8%), eutrophication (3.9%), microplastics (2.9%), and antibiotics (0.9%) were the least investigated topics among the analyzed studies.

#### Studies focused on the recovery of water bodies and correlation with surface waters

**Fig. 6:**
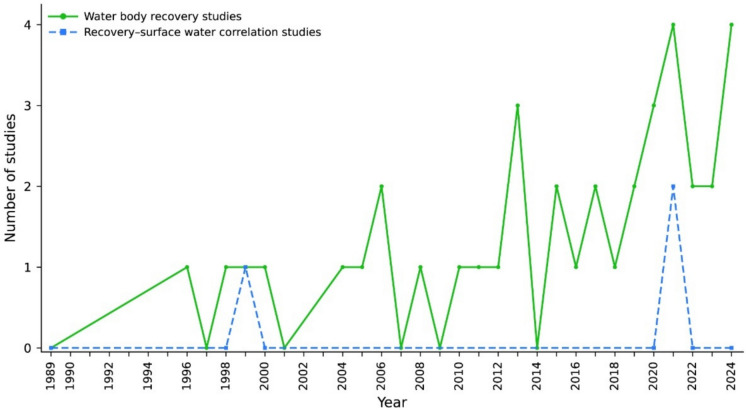
Studies on water body recovery and surface water correlation over time. The graph displays the annual number of publications focusing on water body recovery (green line) and those investigating the correlation between this recovery and surface waters (blue line)

Three investigations involving HZ monitoring focused on water bodies undergoing recovery. The first study was conducted in 1999 and investigated a river recovering from heavy metal contamination (Nelson & Roline, 1999). The other two studies were published in 2021, revealing no clear temporal pattern (Fig. 6). In contrast, studies correlating groundwater investigations with surface waters exhibited a more consistent temporal trend. Between 1995 and 2000, 6.9% of the publications established this correlation. This percentage increased steadily in the subsequent periods: 10.8% from 2001 to 2006, 20.6% from 2007 to 2012, 24.5% from 2013 to 2018, and 36.3% from 2019 to 2024.

### Methodologies employed and sampling effort

#### Analysis of methodology used

**Fig. 7:**
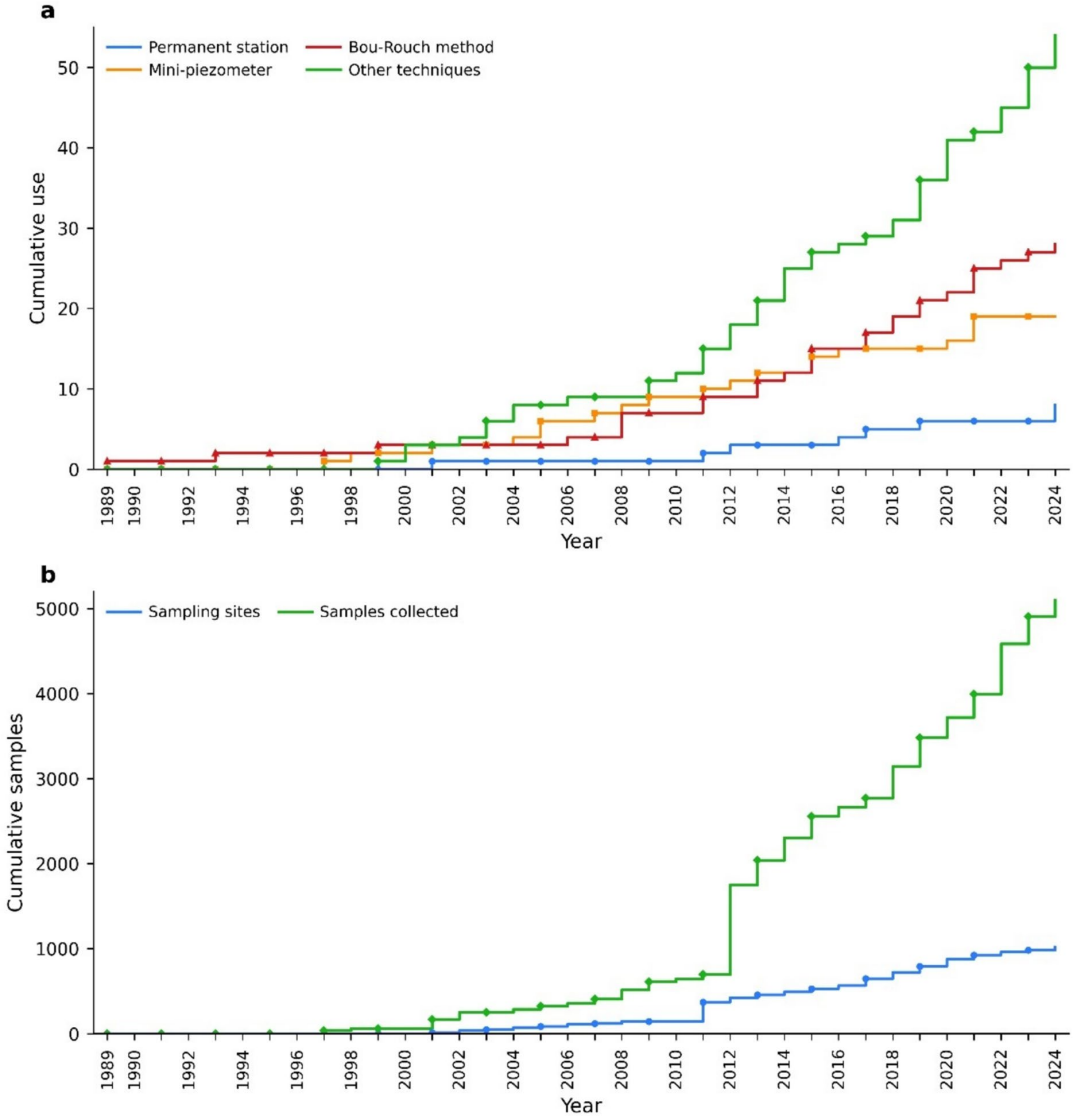
Sampling techniques and effort over time. **a** Usage of various sampling techniques over the years: permanent station (blue), mini-piezometer (orange), Bou-Rouch method (red), and other techniques (green). **b** Temporal trend in sampling effort, illustrating the number of sampling sites (blue) and the number of samples collected (green)

Historically, the first methodology proposed for studying the HZ was the dig pit (Karaman, 1935; Chappuis, 1942), which, despite its ease of execution, does not provide quantitative data. We found no studies employing this methodology. The sampling techniques identified included (1) the Bou-Rouch method, a drilled probe coupled to a suction valve pump (Bou & Rouch, 1967), frequently applied in the version modified by Taglianti et al. (1969) using a lighter and more easily handled membrane pump with lower power (accounting for 25.9% of the conducted studies); (2) mini-piezometers (17.6%), which require a slightly higher time investment but allow for precise temporal analysis, as samples are replicated at the same site over the duration the devices remain in place (Woessner, 2017); and (3) permanent stations (7.4%), used in long-term biomonitoring programs. The “others” category, comprising cores and traps, was proportionally the most utilized (49.0% of the studies; *χ*^2^ = 37.8, *p* < 0.001; Fig. 7a). Considering only studies involving fauna, suction-based sampling techniques (e.g., Bou-Rouch and mini-piezometers) accounted for 43.5% of the applied methods.

We also observed a temporal trend regarding sampling techniques (Fig. 7a). Permanent stations remain rarely used, likely due to high implementation costs. Techniques like the Bou-Rouch method and mini-piezometers exhibited a stationary pattern over the analyzed period. Since 2003, research employing alternative techniques has shown a more pronounced increase.

#### Sampling depth explored in the studies

Regarding sampling depth, there is no clear standardization across studies, indicating that researchers have primarily focused on depths up to − 50 cm. The most frequently sampled depth range was − 30 to − 40 cm, represented in 37.1% of the studies, followed closely by the 0 to − 10 cm (36.1%), − 20 to − 30 cm (35.1%), − 40 to − 50 cm (34.0%), and − 10 to − 20 cm (30.9%) ranges. Notably, there was no significant difference in the frequency of studies among the different 10-cm intervals within the 0 to − 50-cm depth range (*χ*^2^ = 1.1, *p* > 0.05). Depths deeper than − 50 cm were the least explored, appearing in 26.8% of the studies.

#### Sampling effort over time

Analyses of sampling effort were primarily based on the number of samples collected, which accounted for 82.9% of the cases (*χ*^2^ = 43.5, *p* < 0.001). The cumulative number of sampling sites increased steadily over time, with notable increments observed around 2011 and 2012. However, the cumulative number of samples demonstrated a more pronounced growth, particularly from 2012 onward (Fig. 7b).

### Impact of legislation on monitoring activities

#### Effect of the legislation on the number of studies

Specific legislation addressing groundwater protection has been enacted in three regions: the USA (1996), the European Union (2006), and Australia (2007). Across these regions, 95.0% of the studies were published after their respective legislative implementations (*χ*^2^ = 81.0, *p* < 0.001; Online Resource 2, Fig. a–d). When analyzed separately, this trend remained consistent. In the USA, 96.0% of the studies were published after 1996 (*χ*^2^ = 77.4, *p* < 0.001); in the European Union, 96.0% were published after 2006 (*χ*^2^ = 84.6, *p* < 0.001); and in Australia, 75.0% were published after 2007 (*χ*^2^ = 25.0, *p* < 0.001).

Despite these statistically significant results, the actual increase in publications appears modest when considering the absolute numbers. In Australia, only three articles were published by 2024 following the legislation, corresponding to an average of 0.17 articles per year. In the USA, 15 articles were published after 1996, yielding an annual average of 0.54 articles. In the European Union, 50 articles were published after 2006, resulting in an annual average of 2.78 articles.

Notably, Asian countries, represented predominantly by China, exhibited a higher number of publications than both the USA and Australia, despite the absence of specific legislation addressing the hyporheic zone (Online Resource 2, Fig. d).

## Discussion

Our study aimed to provide a comprehensive overview of HZ monitoring research, addressing multiple dimensions to identify key trends, gaps, and future directions. Specifically, we examined bibliometric patterns, assessed global research distribution, and explored spatial and taxonomic biases in HZ studies. Furthermore, we evaluated the influence of environmental legislation, investigated methodological approaches, and analyzed the extent to which HZ monitoring integrates surface–subsurface interactions. Additionally, we assessed research efforts regarding the vertical dimension of the HZ, environmental impacts, and restoration initiatives, differentiating between short- and long-term monitoring strategies.

### Research trends in HZ monitoring

Recent syntheses and bibliometric studies have primarily examined hyporheic research at a broad scope, focusing on conceptual developments and coupled processes (Ward, 2016) and overall publication patterns in the hyporheic literature (e.g., Wang et al., 2025). Meanwhile, other reviews have targeted specific ecological components, such as hyporheic invertebrates (Pascuale et al., 2024). In contrast, our review isolates the subset of studies explicitly addressing hyporheic zone monitoring for environmental quality and conservation status. We quantitatively characterize its growth, outlet dispersion, and the balance between biological and abiological approaches.

Despite the increased interest in the study of the HZ (Boulton et al., 1998; Mugnai et al., 2015a; Pascuale et al., 2024), our findings indicate an initially slow increase in HZ monitoring programs. This delay in acknowledging this critical ecological interface might reflect the broader scientific community’s initial focus on surface waters before recognizing the unique role of the HZ in hydrological and ecological processes. Our study identified a total of 102 articles published between 1989 and December 2024. This is a relatively small number compared to the 271 ecological studies on the HZ found by Pascuale et al. (2024), and it appears even more modest when juxtaposed with the extensive bodies of literature in related fields, such as surface or marine waters (Machuca-Sepúlveda et al., 2023; Roveta et al., 2021). Thus, while demonstrating growing interest, the volume of HZ monitoring research remains limited.

Our findings highlighted the fragmented nature of research into the HZ, with efforts spread across various disciplines and journals. However, the concentration of 25.5% of the articles in just four journals suggests that, although the scopes of these journals are diverse, certain outlets have become key platforms for disseminating HZ research (Ward, 2016). This pattern reflects a growing but still niche area of study that has yet to achieve the consolidation seen in more mature fields.

The steady increase in publications over time, as indicated by our data, mirrors trends observed in other emerging environmental research areas. This growth is likely driven by the increasing recognition of the HZ’s role in supporting biodiversity (Wood et al., 2012) and nutrient cycling (Bardini et al., 2012), as well as its function as a reservoir for pollutants and pathogens (Kumar et al., 2020; Mugnai et al., 2015b). However, the lack of a clear trend between studies focused on biological versus non-biological data suggests that the research community has not yet settled on a dominant methodological approach. This variability could be seen as a strength, reflecting the interdisciplinary nature of HZ studies, but it also highlights the need for standardized methodologies to facilitate comparisons and synthesize findings across studies.

### Geographic distribution and regional patterns of studies

To our knowledge, no previous review (e.g., Wang et al., 2025) has quantified research trends in HZ monitoring using a monitoring-specific framework comparable to ours. For instance, Wang et al. (2025) assessed productivity and collaboration across the full HZ literature at the country and author affiliation level, where the USA dominates as a single major contributor. In contrast, our PRISMA-screened dataset targets HZ monitoring practice and reports patterns by study region, with Europe leading. These findings are therefore complementary, illustrating that overall hyporheic research and monitoring-focused studies show different geographic signatures due to scope and analytical definitions.

Specifically, our analysis reveals that Europe leads in HZ monitoring studies, followed by Asia and then North America. This trend likely reflects Europe’s long-standing investment in environmental monitoring networks and research infrastructure dedicated to freshwater ecosystems (e.g., Cesarini et al., 2025). The presence of well-funded environmental protection agencies and structured monitoring programs has further facilitated the integration of HZ monitoring into broader water resource management strategies (e.g., Strona et al., 2019).

In contrast, Central and South America, Oceania, and Africa show much lower representation in HZ monitoring research. This underrepresentation may be due to fewer research institutions focused on long-term ecological monitoring, limited funding, and the absence of environmental policies specifically targeting HZ conservation (Kuemmerlen et al., 2022). Additionally, economic and climatic challenges can restrict the establishment of consistent monitoring efforts, particularly in arid or semiarid regions (DelVecchia et al., 2022; Merigó Lindahl et al., 2016).

Interestingly, Asia, particularly China, emerges as a significant contributor to HZ monitoring studies, slightly surpassing North America. This pattern contrasts with expectations based on the continent’s relatively recent focus on HZ monitoring and the lack of legislation specific to the HZ. China’s increasing engagement appears to be driven by the urgent need to address freshwater management challenges associated with rapid urbanization and industrialization, stimulating greater attention to groundwater and surface water interactions (e.g., Ji et al., 2020; Yan et al., 2020; Yang et al., 2014).

### Taxonomic focus and ecological classification

The taxonomic distribution observed in HZ monitoring studies suggests potential methodological and ecological biases. While most studies focus on the zoological community, it is surprising that crustaceans and insects, which are ecologically significant and commonly used in surface water monitoring (Bonacina et al., 2023; Bonada et al., 2006; Issartel & Marmonier, 2025; Rinderhagen et al., 2000), account for only 27.6% of the research. This low percentage is not due to a lack of interest, but rather to the fact that the “other” category, which includes microorganisms such as bacteria and viruses, is gaining increasing attention (Retter et al., 2021). This shift suggests a growing recognition of the HZ’s role as an area of ecosystem service provision (Mammola et al., 2025). Regarding the utilization of trait-based analyses, this approach aims to provide rapid and efficient ecological assessments by minimizing taxonomic identification challenges (Menezes et al., 2010). However, similar to trends observed in surface water studies, this strategy is not widely applied in HZ monitoring, representing only 6.6% of the publications in our dataset. This limited use highlights a significant gap in current research, which is primarily driven by a lack of basic ecological information regarding many interstitial groups (Di Lorenzo et al., 2021; Mugnai et al., 2019; Van den Brink et al., 2011).

Our findings also underscore the prominence of research on hyporheic fauna, as initially defined by Orghidan (1959), which is often coupled with epigean species, while only a small fraction of studies examine phreatic and hypogean species. An integrated approach is essential for understanding how surface and subterranean conditions influence hyporheic communities and vice versa (Brunke & Gonser, 1997). In practical terms, environmental policies and monitoring frameworks may need to expand beyond surface waters to include hyporheic and phreatic zones (Saccò et al., 2024). This expansion could lead to more comprehensive conservation measures that acknowledge the ecological functions of these understudied zones.

### Environmental impacts investigated

Existing reviews (e.g., Peralta-Maraver et al., 2018) largely synthesize hyporheic contaminant processes or focus on specific pollutant classes, and broad scientometric analyses map general hyporheic research trends (Wang et al., 2025). Our synthesis explicitly compares stressor categories across HZ monitoring studies and reveals a strong thematic skew toward specific pollutants.

Regarding the investigated impacts, the accumulation of heavy metals is the most frequently studied issue, reflecting concerns about pollution and its effects on water quality (Liang et al., 2022; Liu et al., 2022; Ward, 2016). This focus may be partly due to advances in analytical techniques, which have improved the detection and quantification of these pollutants, making it easier to assess their effects on subterranean water quality (Peter et al., 2019). Additionally, industrial and urban pollution was prominently studied, indicating significant human impacts on the HZ. These publications highlight the detrimental effects of urbanization and industrialization on groundwater and surface water interactions, emphasizing the need for stricter pollution control measures.

Despite the relevance of clogging and impacts associated with agriculture, the relatively low number of studies in these areas suggests that they remain underexplored. Clogging, in particular, can alter hyporheic flow dynamics and impact nutrient cycling, yet few studies have focused on these processes in a detailed manner (e.g., Saavedra Cifuentes et al., 2023). Agricultural impacts on the HZ, including runoff from fertilizers and pesticides (e.g., Li et al., 2023), also require more attention given their potential to influence groundwater contamination and biodiversity.

The growing interest in microplastics reflects the increasing recognition of this emerging environmental threat and its implications for both aquatic ecosystems and human health (Patil et al., 2022; Wang et al., 2021). This emerging issue may soon surpass traditional pollutants in terms of ecological impact, given its persistence in the environment and widespread occurrence (Hoang et al., 2025).

Looking ahead, there is a clear need for more studies exploring the interaction between multiple pollutants, as the synergistic interactions of combined contaminants can generate non-additive effects that are difficult to predict from studies focusing on individual pollutants in hyporheic environments (e.g., Majeed et al., 2024). Further research is also needed regarding the effectiveness of management strategies in mitigating the effects of these pollutants on groundwater and surface water ecosystems, especially in regions with high agricultural activity or industrial contamination.

### Recovery studies and surface water correlations

To our knowledge, previous syntheses discuss hyporheic processes in restoration and groundwater and surface water connectivity, but they generally do not quantify these themes within a monitoring-specific frame (e.g., Hester & Gooseff, 2010). Our PRISMA-screened dataset shows that only three studies focused on water bodies undergoing recovery. The sporadic nature of these studies indicates that the recovery of aquatic systems, particularly those impacted by contaminants, may still be a niche field within HZ research (Wang et al., 2022). This lack of continuous investigation could be attributed to the complexity and long-term nature of recovery processes, as well as the challenges in obtaining consistent data from recovering ecosystems (Stoffers et al., 2026).

In contrast, studies exploring the correlation between groundwater and surface waters show a steady upward trend. This progressive rise reflects a growing interest in understanding interactions between groundwater and surface waters, which may be driven by the increasing need for integrated approaches to water resource management (e.g., Pokhrel et al., 2022). The focus on these correlations also suggests a shift in research objectives from isolated studies of subsurface environments to a more holistic understanding of the entire aquatic ecosystem. Future research could explore the dynamics between surface waters and groundwater, investigating how changes in one compartment influence the other and how these interactions affect ecological processes at various scales (Sabale et al., 2023).

### Methodological approaches

Building on earlier syntheses that charted the growth of interdisciplinary hyporheic research (Ward, 2016), our review adds a method-focused comparison across studies, showing how sampling choices have shifted with research questions and where evidence is still thin. Early methods, such as the dig pit technique, have fallen out of use due to their inability to provide quantitative data (Baxter et al., 2003). The Bou-Rouch suction pump, introduced in 1967 and later modified, remains a popular method. However, mini-piezometers, which allow for more precise and replicable sampling over time (Baxter et al., 2003; Woessner, 2017), have gained traction. The key-added insight from our review is that this methodological “upgrade” has not yet translated into routine long-term monitoring: permanent stations and multi-season time series are still uncommon, likely because installation and maintenance costs remain high, even though low-cost drive-point and multilevel designs have been demonstrated and could lower this barrier (Rivett et al., 2008). However, long-term stations could support studies that aim to capture temporal changes, especially for understanding the effects of seasonal and climate-driven hydrological changes on HZ processes (Staponites et al., 2022). Expanding access to funding or developing lower-cost alternatives might help facilitate such long-term, temporally precise monitoring efforts. The reliance on suction-based sampling methods further underscores this trend toward methods that balance efficiency with temporal accuracy. Notably, interest in other techniques has evolved, especially since 2003, due to increasing attention to ecological assessment approaches incorporating microbial indicators (Sagova-Mareckova et al., 2021) and heavy metal analyses (Ward, 2016).

### Sampling depths and effort over time

The analysis of the depths explored in the studies reveals a relatively uniform distribution among the sampled ranges, with a slight predominance in intermediate depths. This pattern may reflect logistical considerations, as these depths are more accessible compared to deeper or shallower zones, where collection can be technically challenging and require more resources. The lower representation of depths deeper than 50 cm below the bed surface may indicate the need for specialized equipment for deeper sampling, or it may stem from the perception that deeper areas of the HZ possess lower ecological diversity or reduced functional relevance, even though the evidence base remains limited at these depths (Peralta-Maraver et al., 2018).

Regarding sampling effort, the steady cumulative increase in the number of sites and samples over time, particularly after 2012, suggests a growing research interest in HZ dynamics. This intensified sampling effort could be linked to a broader recognition of the HZ’s ecological importance in water quality maintenance, nutrient cycling, and habitat provision (Boulton et al., 1998). However, the observed growth remains concentrated in studies employing conventional depth ranges, indicating that deeper layers of the HZ are still relatively underexplored and may represent an important frontier for future research.

### Impact of legislation on research output

Regarding legislation, its general function is to ensure both obligation and universality within its scope of action, guaranteeing that measures are enforced across the entire community. The analysis of the impact of specific environmental legislation on the number of HZ-related studies reveals that, where such legislation exists, it appears to have significantly boosted research output in the USA and Europe (Foster & Matlock, 2001). The key added insight from our synthesis, however, is that this “legislation signal” is not spatially universal. The spatial distribution of studies (Online Resource 2) shows persistent clustering within a limited set of regions in both the USA and Europe, implying that local funding, infrastructure, and research priorities shape where evidence is actually produced, even under the same overarching legal framework (Voulvoulis et al., 2017). This effect was not observed in Australia, contrary to what might be expected following the implementation of these laws (Grafton, 2019).

In the USA, since the enactment of these laws, research efforts have remained largely restricted to a small portion of the northeastern and western regions, while most of the territory remains unstudied. In Europe, there is a high concentration of studies in the central region (e.g., France, Germany, and England), but vast areas remain unexplored. Surprisingly, Asian countries (e.g., China) exhibit a higher number of publications than the USA and Australia, despite the absence of specific laws protecting the HZ. This suggests that factors other than legislation, such as growing environmental awareness and investment in research, may also drive scientific inquiry in these regions (Higgins et al., 2021; Kuemmerlen et al., 2022).

In summary, although the implementation of specific legislation has been associated with a significant increase in the number of studies on the HZ in certain regions, its overall impact is shaped by multiple contextual factors. The concentration of studies in specific areas, even after the enactment of these laws, indicates that legislation, while necessary, is not sufficient to ensure the widespread development of research efforts. Elements such as research infrastructure, targeted funding, and local science policies appear to play equally important roles (Stefanidis & Papastergiadou, 2024). Furthermore, the example of Asian countries, which has expanded their scientific output in the absence of specific HZ protection laws, highlights the growing influence of broader socioeconomic and environmental factors in shaping research agendas. Therefore, strengthening integrated strategies by combining legislation, funding, the development of research networks, and the promotion of environmental awareness will be essential to broaden and democratize knowledge on the HZ at a global scale.

### A roadmap for advancing HZ monitoring

**Fig. 8:**
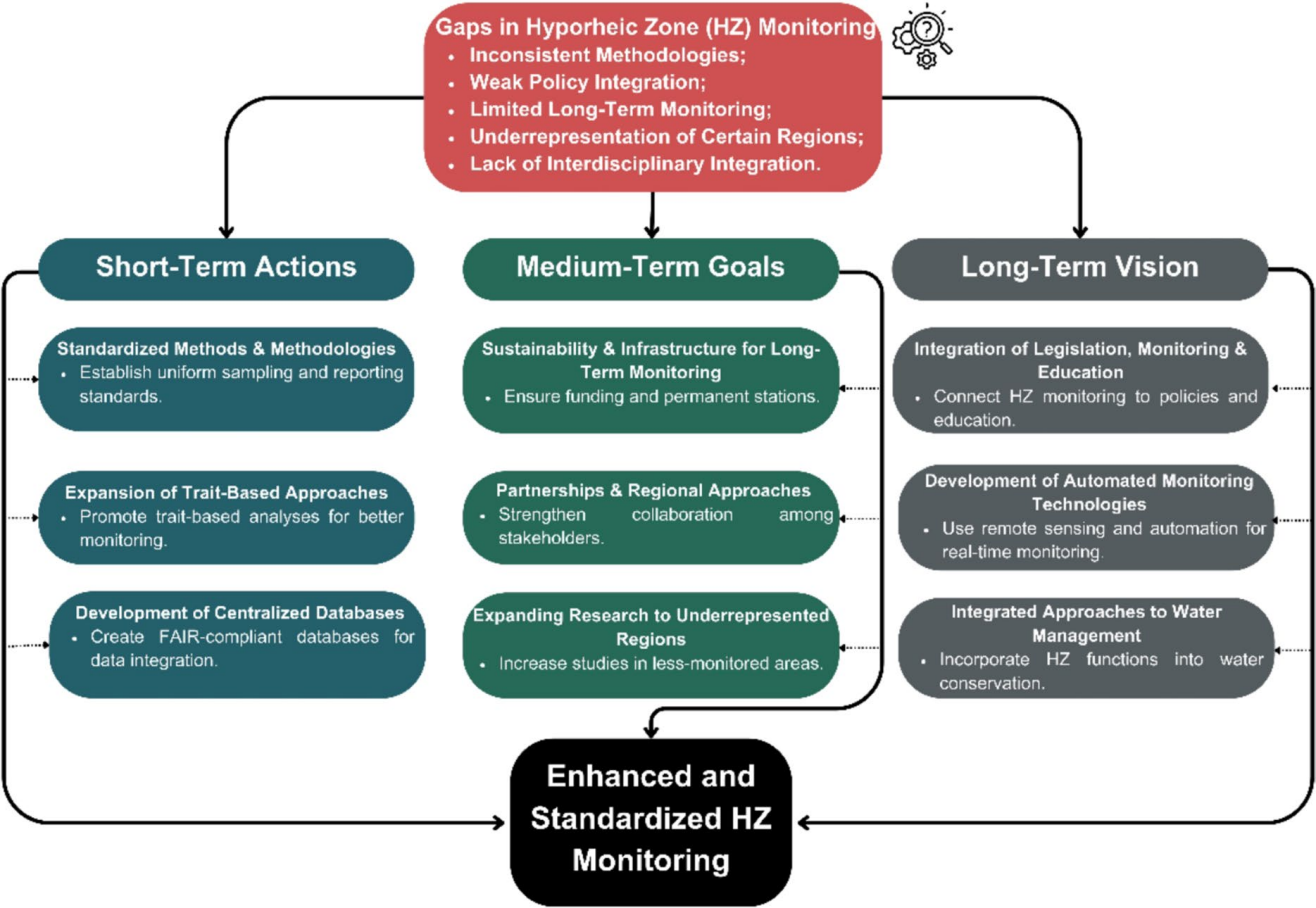
A roadmap combining scientific research and policy efforts to advance hyporheic zone (HZ) monitoring and promote effective water resource management

Despite advances in technological and theoretical knowledge and the awareness that the preservation of water resources is necessary and urgent (Jury & Vaux, 2005), the results of efforts to monitor HZ remain below expectations. Regulations alone, without integrated interventions, have limited impact, and in the case of HZ monitoring, a more strategic approach is needed. This requires fostering partnerships between local governments, research institutions, and communities to develop monitoring strategies that are both context-specific, long-term, and adaptable to regional conditions. Below, we outline a roadmap that combines scientific research and policy efforts to advance HZ monitoring and promote effective water resource management, structured into three main phases: short-term actions, medium-term goals, and a long-term vision (Fig. 8).

### Short-term actions

1. Standardized methods and methodologies

To improve HZ monitoring and facilitate meaningful comparisons across studies, there is an urgent need to standardize methodologies at a broad scale. Establishing uniform protocols for sampling techniques, data analysis, and reporting will enhance the reliability and comparability of research across different regions and disciplines (Behmel et al., 2016). Additionally, identifying key ecological and hydrological questions that remain unaddressed will help direct research efforts toward critical knowledge gaps (Mammola et al., 2020). A unified methodological framework is essential for ensuring consistency in research quality and for enabling large-scale assessments of HZ dynamics.

(2) Expansion of trait-based approaches

The expansion of trait-based approaches to facilitate efficient monitoring is recommended. Trait-based analyses aim to minimize study effort by enabling rapid and efficient ecological assessments while reducing the challenges of taxonomic identification. The trait-based approach to the HZ would also benefit from standardized reporting and best practices for analysis. A multi-step protocol, encompassing everything from defining a research question and assembling data matrices to model fitting and data sharing, would help enhance reproducibility, transparency, and comparability across studies (Palacio et al., 2022).

(3) Development of centralized databases

Establishing centralized databases that adhere to FAIR principles, ensuring data is findable, accessible, interoperable, and reusable, will significantly enhance data accessibility, reproducibility, and integration across diverse research initiatives (Wilkinson et al., 2016). These robust databases will not only support long-term data preservation and seamless sharing but also empower researchers to perform comprehensive meta-analyses, ultimately advancing our understanding of HZ dynamics and fostering collaborative scientific progress (Saccò et al., 2024).

### Medium-term goals

(4) Sustainability and infrastructure for long-term monitoring

Ensuring the long-term success of HZ monitoring efforts requires sustainable funding mechanisms and the development of adequate infrastructure. Many monitoring initiatives fail due to short-term funding cycles and the lack of institutional support (Adamo et al., 2022). To address this, governments and research organizations must prioritize long-term investments in monitoring programs, securing financial resources that allow for continuous data collection and analysis. Establishing permanent monitoring stations and investing in technological advancements will ensure that HZ studies remain consistent and scientifically robust over time.

(5) Partnerships and regional approaches

One of the main obstacles to effective HZ monitoring is the lack of coordinated efforts between different sectors and stakeholders. Addressing these challenges requires strong partnerships between local governments, research institutions, and community stakeholders. These collaborations are crucial for developing monitoring strategies tailored to specific regional needs and conditions, considering the unique environmental, social, and economic factors of each location (Ban et al., 2018). Locally driven approaches not only enhance the relevance and applicability of research but also ensure the feasibility of implementation in resource-limited areas. Engaging local actors, including citizen scientists (Schneider et al., 2023), allows for better data collection, knowledge sharing, and policy alignment, increasing the chances of long-term success (Kobori et al., 2016). By leveraging local expertise and fostering cooperation, monitoring efforts can become more effective, cost-efficient, and sustainable in the long term.

(6) Expanding research to underrepresented regions

HZ research is disproportionately concentrated in certain areas, leaving vast regions, such as Central and South America, Oceania, and Africa, significantly underrepresented. Expanding research in these regions is essential for achieving a more global perspective on HZ functions and their role in water resource management (Koch et al., 2024). This expansion requires increased funding accessibility, stronger local collaborations, and the development of international partnerships that facilitate knowledge exchange and capacity building (Krause et al., 2011). By broadening research coverage, we can develop more inclusive and representative conservation strategies.

### Long-term vision

(7) Integration of legislation, monitoring, and education

The effectiveness of environmental legislation depends on its integration with active monitoring programs and public engagement initiatives (Lawrence et al., 2013). Additionally, public education plays a crucial role in raising awareness about the importance of the HZ and its functions within aquatic ecosystems. Educational initiatives should be designed to engage local communities, policymakers, and industry stakeholders, fostering a culture of conservation and responsible water resource management (Kobori et al., 2016).

(8) Development of automated monitoring technologies

Advancements in automated monitoring technologies, such as remote sensing, machine learning applications, and advanced in situ sensor networks, can enhance the efficiency of HZ monitoring (Shaikh & Birajdar, 2024; Thomle et al., 2020). These tools enable continuous, real-time monitoring of critical environmental parameters, improving data accuracy and collection efficiency (Zhou et al., 2018). Integrating these technologies supports adaptive management strategies, contributing to more effective water resource management.

(9) Integrated approaches to water management

Water resource management must adopt a holistic approach that incorporates ecosystem services and hydrological functions into decision-making processes (Canedoli et al., 2022). The HZ plays a fundamental role in water filtration, nutrient cycling, and habitat provision, yet it is often overlooked in traditional water management frameworks. Integrating HZ functions into water policy and management strategies will lead to more sustainable and effective conservation efforts (Krause et al., 2011). By promoting interdisciplinary collaboration and evidence-based policies, we can enhance the resilience of water systems and improve long-term water security (Boulton et al., 2010).

To implement this roadmap, we suggest a staged pathway that directly supports monitoring, modeling, and management. In the short term, agencies can pilot a small “HZ module” within routine river surveys, using harmonized protocols plus a minimum set of shared metadata (e.g., depth, hydraulic gradients, sediment context, basic chemistry) so results become comparable across sites and programs (Ward, 2016). In the medium term, these standardized datasets can be used to calibrate and test hyporheic exchange and transport models, with an emphasis on exchange fluxes and residence times, and on uncertainty reporting that is usable for decisions (Boano et al., 2014). Finally, in the long term, hyporheic indicators can be embedded as performance metrics for restoration and pollution mitigation, using simple before-and-after designs to track the recovery of vertical connectivity and ecosystem services (Boulton, 2007).

### Study limitations

Although this review provides a broad overview of global HZ monitoring, specific methodological limitations must be acknowledged. First, our literature search was restricted to major scientific databases and peer-reviewed articles published in English. This approach inherently excludes gray literature, such as technical reports from environmental protection agencies, as well as regional studies published in other languages, which could contain local monitoring data. Furthermore, there is a geographical bias in the retrieved literature, as most of the monitoring data comes from Europe and North America. This concentration affects the overall representativeness of the findings. Consequently, the ecological patterns and methodologies discussed here might not fully translate to understudied areas. Regions like Central and South America, Africa, and Oceania have different hydrological dynamics, climates, and biodiversity profiles compared to the Northern Hemisphere. Therefore, expanding monitoring efforts in these regions would be a valuable step toward a more balanced understanding of HZ management worldwide.

## Conclusion

This systematic review provides the first global synthesis of monitoring practices in the hyporheic zone, revealing important structural imbalances and knowledge gaps. While our study reveals a steady increase in HZ-related studies, the field remains fragmented, with research efforts dispersed across multiple disciplines and largely confined to a few dominant journals. Our analysis also shows that monitoring efforts are geographically uneven and predominantly focused on physicochemical and hydrological parameters, while biological indicators, despite their recognized ecological relevance, remain underrepresented. Methodological heterogeneity in sampling depth, temporal resolution, sediment characterization, and taxonomic identification further limits cross-study comparability and the development of harmonized assessment frameworks. Many studies are site-specific and short-term, restricting the ability to generalize findings across climatic and geomorphological contexts. Inconsistent methodological reporting and the absence of standardized protocols complicate meta-comparisons and reduce reproducibility. Moreover, limited integration of biological, chemical, and hydrological components constrains a process-based understanding of ecosystem functioning and pollutant dynamics in the HZ. From a management perspective, these biases matter because they create blind spots: the predominance of heavy metal studies, alongside the relative neglect of emerging pollutants, such as microplastics, points to critical monitoring gaps that risk forcing managers into reactive responses only after degradation is advanced. A key insight from our analysis is that legislative measures alone have not significantly driven research output. Although countries with specific HZ regulations have produced more studies than those without, the overall effect remains marginal. This suggests that policy frameworks must be complemented by institutional support, funding mechanisms, and interdisciplinary collaboration to translate legal mandates into meaningful research efforts.

Moving forward, achieving a more comprehensive and effective HZ monitoring strategy requires a coordinated roadmap that integrates scientific advancements, policy development, and sustainable long-term monitoring efforts. Future research should prioritize (1) the development of standardized monitoring protocols, (2) long-term and multi-site assessments across underrepresented regions, (3) stronger integration of biological indicators with hydrological and chemical measurements, and (4) the incorporation of biological feedback mechanisms into predictive models of solute transport and contaminant processing. Interdisciplinary approaches linking ecohydrology, microbiology, and environmental modeling will be essential to advance mechanistic understanding.

From a management perspective, incorporating the HZ explicitly into environmental monitoring frameworks and legislation, and adopting ecosystem-based approaches, will enhance the effectiveness of targeted interventions. For instance, incorporating biological indicators, such as microbial community structure, biofilm development, and hyporheic invertebrate assemblages, into routine monitoring would enhance the early detection of impairment of the ecosystem services they provide. By addressing these challenges, future hyporheic zone research can evolve from an isolated research focus into a cornerstone of global water resource management.

## Supplementary Information

Below is the link to the electronic supplementary material.

ESM 1 (XLSX 185 KB)

ESM 2 (PDF 183 KB)

## Data Availability

Data will be made available on request.

## References

[CR1] Adamo, M., Sousa, R., Wipf, S., Correia, R. A., Lumia, A., Mucciarelli, M., & Mammola, S. (2022). Dimension and impact of biases in funding for species and habitat conservation. *Biological Conservation,**272*, Article 109636. 10.1016/j.biocon.2022.109636

[CR2] Ban, N. C., Frid, A., Reid, M., Edgar, B., Shaw, D., & Siwallace, P. (2018). Incorporate indigenous perspectives for impactful research and effective management. *Nature Ecology & Evolution,**2*(11), 1680–1683. 10.1038/s41559-018-0706-0 30349090 10.1038/s41559-018-0706-0

[CR3] Bardini, L., Boano, F., Cardenas, M. B., Revelli, R., & Ridolfi, L. (2012). Nutrient cycling in bedform induced hyporheic zones. *Geochimica Et Cosmochimica Acta,**84*, 47–61. 10.1016/j.gca.2012.01.025

[CR4] Baxter, C., Hauer, F. R., & Woessner, W. W. (2003). Measuring groundwater–stream water exchange: New techniques for installing minipiezometers and estimating hydraulic conductivity. *Transactions of the American Fisheries Society,**132*(3), 493–502. 10.1577/1548-8659(2003)132<0493:MGWENT>2.0.CO;2

[CR5] Behmel, S., Damour, M., Ludwig, R., & Rodriguez, M. J. (2016). Water quality monitoring strategies—A review and future perspectives. *Science of the Total Environment,**571*, 1312–1329. 10.1016/j.scitotenv.2016.06.235 27396312 10.1016/j.scitotenv.2016.06.235

[CR6] Birk, S., Chapman, D., Carvalho, L., Spears, B. M., Andersen, H. E., Argillier, C., Auer, S., Baattrup-Pedersen, A., Banin, L., Beklioğlu, M., Bondar-Kunze, E., Borja, A., Branco, P., Bucak, T., Buijse, A. D., Cardoso, A. C., Couture, R.-M., Cremona, F., de Zwart, D., … Hering, D. (2020). Impacts of multiple stressors on freshwater biota across spatial scales and ecosystems. *Nature Ecology & Evolution,**4*(8), 1060–1068. 10.1038/s41559-020-1216-4 32541802 10.1038/s41559-020-1216-4

[CR7] Boano, F., Harvey, J. W., Marion, A., Packman, A. I., Revelli, R., Ridolfi, L., & Wörman, A. (2014). Hyporheic flow and transport processes: Mechanisms, models, and biogeochemical implications. *Reviews of Geophysics,**52*(4), 603–679. 10.1002/2012RG000417

[CR8] Bonacina, L., Fasano, F., Mezzanotte, V., & Fornaroli, R. (2023). Effects of water temperature on freshwater macroinvertebrates: A systematic review. *Biological Reviews,**98*(5), 1621–1639. 10.1111/brv.12971 10.1111/brv.12903 PMC10088029 36173002

[CR9] Bonada, N., Prat, N., Resh, V. H., & Stetzer, B. (2006). Developments in aquatic insect biomonitoring: A comparative analysis of recent approaches. *Annual Review of Entomology,**51*(1), 495–523. 10.1146/annurev.ento.51.110104.151124 10.1146/annurev.ento.51.110104.151124 16332221

[CR10] Boretti, A., & Rosa, L. (2019). Reassessing the projections of the World Water Development Report. *Npj Clean Water,**2*(1), Article 15. 10.1038/s41545-019-0039-9

[CR11] Borsetto, C., Dykes, C., Kockiri, B., Song, L., Wellington, E. M. H., & Abolfathi, S. (2025). Constructed wetlands as nature-based barriers: Mitigating antimicrobial resistance and pathogen dispersal in riverine systems. *Journal of Hazardous Materials,**495*, Article 138855. 10.1016/j.jhazmat.2025.138855 40505399 10.1016/j.jhazmat.2025.138855

[CR12] Bou, C., & Rouch, R. (1967). Un nouveau champ de recherches sur la faune aquatique souterraine. *Comptes Rendus De L’ Academie des Sciences Serie IIa: Sciences De La Terre Et des Planets,**265*, 369–370.

[CR13] Boulton, A. J. (2007). Hyporheic rehabilitation in rivers: Restoring vertical connectivity. *Freshwater Biology,**52*(4), 632–650. 10.1111/j.1365-2427.2006.01710.x

[CR14] Boulton, A. J., Findlay, S., Marmonier, P., Stanley, E. H., & Valett, H. M. (1998). The functional significance of the hyporheic zone in streams and rivers. *Annual Review of Ecology and Systematics,**29*(1), 59–81. 10.1146/annurev.ecolsys.29.1.59

[CR15] Boulton, A., Harvey, M., & Proctor, H. (2004). Of spates and species: Responses by interstitial water mites to simulated spates in a subtropical Australian river. *Experimental and Applied Acarology,**34*, 149–169. 10.1007/978-94-017-0429-8_11 15597606 10.1023/b:appa.0000044445.30246.b2

[CR16] Boulton, A. J., Datry, T., Kasahara, T., Mutz, M., & Stanford, J. A. (2010). Ecology and management of the hyporheic zone: Stream–groundwater interactions of running waters and their floodplains. *Journal of the North American Benthological Society,**29*(1), 26–40. 10.1899/08-017.1

[CR17] Brunke, M., & Gonser, T. (1997). The ecological significance of exchange processes between rivers and groundwater. *Freshwater Biology,**37*(1), 1–33. 10.1046/j.1365-2427.1997.00143.x

[CR18] Canedoli, C., Ficetola, G. F., Corengia, D., Tognini, P., Ferrario, A., & Padoa-Schioppa, E. (2022). Integrating landscape ecology and the assessment of ecosystem services in the study of karst areas. *Landscape Ecology,**37*, 347–365. 10.1007/s10980-021-0135-2

[CR19] Cesarini, G., Fornaroli, R., Boggero, A., Musazzi, S., Zaupa, S., Dumnicka, E., Marchetto, A., & Rogora, M. (2025). First assessment of freshwater monitoring under the EU national emission ceilings directive: Emerging issues and way forward. *Water, Air, & Soil Pollution,**236*(3), Article 181. 10.1007/s11270-025-07804-7

[CR20] Cook, S., Price, O. R., King, A., Finnegan, C., van Egmond, R. A., Schäfer, H., Pearson, J. M., Abolfathi, S., & Bending, G. D. (2020). Bedform characteristics and biofilm community development interact to modify hyporheic exchange. *Science of the Total Environment,**749*, Article 141397. 10.1016/j.scitotenv.2020.141397 32841855 10.1016/j.scitotenv.2020.141397

[CR21] Datry, T., & Larned, S. T. (2008). River flow controls ecological processes and invertebrate assemblages in subsurface flowpaths of an ephemeral river reach. *Canadian Journal of Fisheries and Aquatic Sciences,**65*(8), 1532–1544. 10.1139/F08-075

[CR22] DelVecchia, A. G., Shanafield, M., Zimmer, M. A., Busch, M. H., Krabbenhoft, C. A., Stubbington, R., Kaiser, K. E., Burrows, R. M., Hosen, J., Datry, T., Kampf, S. K., Zipper, S. C., Fritz, K., Costigan, K., & Allen, D. C. (2022). Reconceptualizing the hyporheic zone for nonperennial rivers and streams. *Freshwater Science,**41*(2), 167–182. 10.1086/720071 35846249 10.1086/720071 PMC9280706

[CR23] Di Lorenzo, T., Fiasca, B., Di Cicco, M., Cifoni, M., & Galassi, D. M. (2021). Taxonomic and functional trait variation along a gradient of ammonium contamination in the hyporheic zone of a Mediterranean stream. *Ecological Indicators,**132*, Article 108268. 10.1016/j.ecolind.2021.108268

[CR24] Di Lorenzo, T., Lunghi, E., Aanei, C., Altermatt, F., Alther, R., Amorim, I. R., Băncilă, R. I., Bellvert, A., Blomberg, A., Borges, P. A. V., Brad, T., Brancelj, A., Brankovits, D., Cardoso, P., Cerasoli, F., Chauveau, C. A., Crespo, L., Csader, M., Delić, T., & Mammola, S. (2024). EU needs groundwater ecosystems guidelines. *Science,**386*(6726), 1103. 10.1126/science.ads8140 39636973 10.1126/science.ads8140

[CR25] Dykes, C., Pearson, J. M., Bending, G. D., & Abolfathi, S. (2025). Impact of seasonal climate variability on constructed wetland treatment efficiency. *Journal of Water Process Engineering,**72*, Article 107350. 10.1016/j.jwpe.2025.107350

[CR26] European Community. (2006). Directive 2006/118/EC of the European Parliament and of the Council of 12 December 2006 on the protection of groundwater against pollution and deterioration. *Official Journal of the European Union*, *372*, 19–31.

[CR27] Environmental Protection Authority. (2016). *Technical guidance: Subterranean fauna surveys for environmental impact assessment* (Environmental Assessment Guideline No. 12). Government of Western Australia.

[CR28] Findlay, S. (1995). Importance of surface-subsurface exchange in stream ecosystems: The hyporheic zone. *Limnology and Oceanography,**40*(1), 159–164. 10.4319/lo.1995.40.1.0159

[CR29] Foster, C. A., & Matlock, M. D. (2001). History of the Clean Water Act. *Water Resources Impact,**3*(5), 26–30.

[CR30] Gibert, J. (1991). Groundwater systems and their boundaries: Conceptual framework and prospects in groundwater ecology. *Verhandlungen der Internationalen Vereinigung Für Theoretische und Angewandte Limnologie,**24*(3), 1605–1608. 10.1080/03680770.1989.11899029

[CR31] Grafton, R. Q. (2019). Policy review of water reform in the Murray–Darling Basin, Australia: The “do’s” and “do’nots.” *Australian Journal of Agricultural and Resource Economics,**63*(1), 116–141. 10.1111/1467-8489.12288

[CR32] Hancock, P. J. (2002). Human impacts on the stream–groundwater exchange zone. *Environmental Management,**29*, 763–781. 10.1007/s00267-001-0064-5 11992170 10.1007/s00267-001-0064-5

[CR33] Hester, E. T., & Gooseff, M. N. (2010). Moving beyond the banks: Hyporheic restoration is fundamental to restoring ecological services and functions of streams. *Environmental Science & Technology,**44*(5), 1521–1525. 10.1021/es902988n 20131901 10.1021/es902988n

[CR34] Higgins, J., Zablocki, J., Newsock, A., Krolopp, A., Tabas, P., & Salama, M. (2021). Durable freshwater protection: A framework for establishing and maintaining long-term protection for freshwater ecosystems and the values they sustain. *Sustainability,**13*(4), Article 1950. 10.3390/su13041950

[CR35] Hoang, V. H., Nguyen, M. K., Hoang, T. D., Rangel-Buitrago, N., Lin, C., Pham, M. T., Ha, M. C., Nguyen, T. P., Shaaban, M., Chang, S. W., & Nguyen, D. D. (2025). Microplastic characteristics, transport, risks, and remediation in groundwater: A review. *Environmental Chemistry Letters,**23*(3), 817–837. 10.1007/s10311-025-01825-8

[CR36] Issartel, C., & Marmonier, P. (2025). Description and use of *Schellencandona rhodanensis* sp. n. (Ostracoda, Candoninae) to locate groundwater upwelling zones in rivers and wetlands. *International Journal of Limnology*. 10.1051/limn/2025001

[CR37] Ji, Y., Zhang, J., Zhang, H., Liu, X. C., Wu, N. C., & Cai, G. T. (2020). Review on hotspots, challenges, and the future of river management strategies in China. *Journal of Environmental Biology,**41*(1), 13–22. 10.22438/jeb/41/1/MRN-112

[CR38] Jury, W. A., & Vaux, H., Jr. (2005). The role of science in solving the world's emerging water problems. *Proceedings of the National Academy of Sciences*, *102*(44), 15715–15720. 10.1073/pnas.0506467102 10.1073/pnas.0506467102 PMC1276073 16249337

[CR39] Karaman, S. (1935). Die Fauna der unterirdischen Gewässer Jugoslawiens. *Verhandlungen der Internationalen Vereinigung Für Limnologie,**7*(1), 46–73. 10.1080/03680770.1935.11902405

[CR40] Kobori, H., Dickinson, J. L., Washitani, I., Sakurai, R., Amano, T., Komatsu, N., Kitamura, W., Takagawa, S., Koyama, K., Ogawara, T., & Miller-Rushing, A. J. (2016). Citizen science: A new approach to advance ecology, education, and conservation. *Ecological Research,**31*, 1–19. 10.1007/s11284-015-1314-y

[CR41] Koch, F., Blum, P., Korbel, K., & Menberg, K. (2024). Global overview on groundwater fauna. *Ecohydrology,**17*(1), Article e2607. 10.1002/eco.2607

[CR42] Krause, S., Hannah, D. M., Fleckenstein, J. H., Heppell, C. M., Kaeser, D., Pickup, R., Pinay, G., Robertson, A. L., & Wood, P. J. (2011). Inter‐disciplinary perspectives on processes in the hyporheic zone. *Ecohydrology,**4*(4), 481–499. 10.1002/eco.176

[CR43] Kuemmerlen, M., Batista-Morales, A. M., Bruder, A., Turak, E., & de Oliveira Roque, F. (2022). Conservation of Latin America freshwater biodiversity: Beyond political borders. *Biodiversity and Conservation,**31*(4), 1427–1433. 10.1007/s10531-022-02380-2

[CR44] Kumar, M., Thakur, A. K., Mazumder, P., Kuroda, K., Mohapatra, S., Rinklebe, J., Ramanathan, Z., Cetecioglu, Z., Jain, S., Tyagi, V. K., Gikas, P., Chakraborty, S., Islam, M. T., Ahmad, A., Shah, A. V., Patel, A. K., Watanabe, T., Vithanage, M., Bibby, K., & Bhattacharya, P. (2020). Frontier review on the propensity and repercussion of SARS-CoV-2 migration to aquatic environment. *Journal of Hazardous Materials Letters,**1*, 100001. 10.1016/j.hazl.2020.100001 34977840 10.1016/j.hazl.2020.100001 PMC7456799

[CR45] Lafont, M., Camus, J. C., & Rosso, A. (1996). Superficial and hyporheic oligochaete communities as indicators of pollution and water exchange in the River Moselle, France. *Hydrobiologia,**334*, 147–155. 10.1007/BF00017364

[CR46] Lawrence, J. E., Skold, M. E., Hussain, F. A., Silverman, D. R., Resh, V. H., Sedlak, D. L., Luthy, R. G., & McCray, J. E. (2013). Hyporheic zone in urban streams: A review and opportunities for enhancing water quality and improving aquatic habitat by active management. *Environmental Engineering Science,**30*(8), 480–501. 10.1089/ees.2012.0235

[CR47] Lewandowski, J., Arnon, S., Banks, E., Batelaan, O., Betterle, A., Broecker, T., Coll, C., Drummond, J. D., Gaona Garcia, J., Galloway, J., Gomez-Velez, J., Grabowski, R. C., Herzog, S. P., Hinkelmann, R., Höhne, A., Hollender, J., Horn, M. A., Jaeger, A., Krause, S., & Wu, L. (2019). Is the hyporheic zone relevant beyond the scientific community? *Water,**11*(11), 2230. 10.3390/w11112230

[CR48] Li, S., Arnscheidt, J., Cassidy, R., Douglas, R. W., McGrogan, H. J., & Jordan, P. (2023). The spatial and temporal dynamics of sediment phosphorus attenuation and release in impacted stream catchments. *Water Research,**245*, Article 120663. 10.1016/j.watres.2023.120663 37774540 10.1016/j.watres.2023.120663

[CR49] Liang, D., Song, J., Xia, J., Chang, J., Kong, F., Sun, H., Wu, Q., Cheng, D., & Zhang, Y. (2022). Effects of heavy metals and hyporheic exchange on microbial community structure and functions in hyporheic zone. *Journal of Environmental Management,**303*, Article 114201. 10.1016/j.jenvman.2021.114201 34861506 10.1016/j.jenvman.2021.114201

[CR50] Liu, R., Liu, F., Jiao, J., Xu, Y., Dong, Y., El-Wardany, R. M., Zhang, X., & Chen, H. (2022). Potential toxic impacts of Hg migration in the disjointed hyporheic zone in the gold mining area experiencing river water level changes. *Water,**14*(19), Article 2950. 10.3390/w14192950

[CR51] Machuca-Sepúlveda, J., Miranda, J., Lefin, N., Pedroso, A., Beltrán, J. F., & Farias, J. G. (2023). Current status of omics in biological quality elements for freshwater biomonitoring. *Biology,**12*(7), Article 923. 10.3390/biology12070923 37508354 10.3390/biology12070923 PMC10376755

[CR52] Majeed, L. R., Majeed, L. F., Rashid, S., Bhat, S. A., Kumar, N., & Kumar, V. (2024). Intensification of contaminants, hydrology, and pollution of hyporheic zone: The liver of river ecology—a review. *Environmental Sustainability,**7*(2), 121–133. 10.1007/s42398-023-00290-9

[CR53] Malard, F., Tockner, K., Dole‐Olivier, M. J., & Ward, J. V. (2002). A landscape perspective of surface–subsurface hydrological exchanges in river corridors. *Freshwater Biology,**47*(4), 621–640. 10.1046/j.1365-2427.2002.00906.x

[CR54] Mammola, S., Amorim, I. R., Bichuette, M. E., Borges, P. A. V., Cheeptham, N., Cooper, S. J. B., Culver, D. C., Deharveng, L., Eme, D., Ferreira, R. L., Fišer, C., Fišer, Ž, Fong, D. W., Griebler, C., Jeffery, W. R., Jugovic, J., Kowalko, J. E., Lilley, T. M., Malard, F., … Cardoso, P. (2020). Fundamental research questions in subterranean biology. *Biological Reviews,**95*(6), 1855–1872. 10.1111/brv.12642 32841483 10.1111/brv.12642

[CR55] Mammola, S., Brankovits, D., Di Lorenzo, T., Amorim, I. R., Bancila, R. I., Bellvert, A., ... & Martínez, A. (2025). Subterranean environments contribute to three‐quarters of classified ecosystem services. *Biological Reviews*. Advance online publication.10.1002/brv.70137 10.1002/brv.70137 PMC13149732 41665280

[CR56] Menezes, S., Baird, D. J., & Soares, A. M. (2010). Beyond taxonomy: A review of macroinvertebrate trait‐based community descriptors as tools for freshwater biomonitoring. *Journal of Applied Ecology,**47*(4), 711–719. 10.1111/j.1365-2664.2010.01819.x

[CR57] Merigó Lindahl, J., Cancino del Castillo, C., Coronado Martínez, F., & Urbano, D. (2016). Academic research in innovation: A country analysis. *Scientometrics,**108*, 559–593. 10.1007/s11192-016-1984-4

[CR58] Moghaddam, H. K., Kivi, Z. R., Abtahizadeh, E., & Abolfathi, S. (2025). Sustainable water allocation under climate change: Deep learning approaches to predict drinking water shortages. *Journal of Environmental Management,**385*, Article 125600. 10.1016/j.jenvman.2025.125600 40345087 10.1016/j.jenvman.2025.125600

[CR59] Mohammadpour, A., Gharehchahi, E., Golaki, M., Gharaghani, M. A., Ahmadian, F., Abolfathi, S., Samaei, M. R., Uddin, M. G., Olbert, A. I., & Khaneghah, A. M. (2025). Advanced water quality assessment using machine learning: Source identification and probabilistic health risk analysis. *Results in Engineering,**27*, Article 105421. 10.1016/j.rineng.2025.105421

[CR60] Mugnai, R., Messana, G., & Di Lorenzo, T. (2015a). The hyporheic zone and its functions: Revision and research status in Neotropical regions. *Brazilian Journal of Biology,**75*(3), 524–534. 10.1590/1519-6984.15413 10.1590/1519-6984.15413 26421769

[CR61] Mugnai, R., Sattamini, A., Albuquerque dos Santos, J. A., & Regua-Mangia, A. H. (2015b). A survey of *Escherichia coli* and *Salmonella* in the hyporheic zone of a subtropical stream: Their bacteriological, physicochemical and environmental relationships. *PLoS ONE,**10*(6), Article e0129382. 10.1371/journal.pone.0129382 26067288 10.1371/journal.pone.0129382 PMC4466359

[CR62] Mugnai, R., Serpa-Filho, A., Nessimian, J. L., Kury, A. B., & Milesi, S. V. (2019). Morphological traits and vertical distribution of hyporheic chironomid larvae in Atlantic Forest streams. *Tropical Zoology,**32*(3), 119–134. 10.1080/03946975.2019.1639034

[CR63] Nelson, S. M., & Roline, R. A. (1999). Relationships between metals and hyporheic invertebrate community structure in a river recovering from metals contamination. *Hydrobiologia,**397*, 211–226. 10.1023/A:1003734407788

[CR64] Nogaro, G., Datry, T., Mermillod-Blondin, F., Descloux, S., & Montuelle, B. (2010). Influence of streambed sediment clogging on microbial processes in the hyporheic zone. *Freshwater Biology,**55*(6), 1288–1302. 10.1111/j.1365-2427.2009.02352.x

[CR65] Orghidan, T. (1959). Ein neuer Lebensraum des unterirdischen Wassers: Der hyporheische Biotop. *Archiv Für Hydrobiologie,**55*(3), 392–414.

[CR66] Orghidan, T. (2010). A new habitat of subsurface waters: The hyporheic biotope. *Fundamental and Applied Limnology,**176*(4), 291.

[CR67] Page, M. J., McKenzie, J. E., Bossuyt, P. M., Boutron, I., Hoffmann, T. C., Mulrow, C. D., Shamseer, L., Tetzlaff, J. M., Akl, E. A., Brennan, S. E., Chou, R., Glanville, J., Grimshaw, J. M., Hróbjartsson, A., Lalu, M. M., Li, T., Loder, E. W., Mayo-Wilson, E., McDonald, S., ... Moher, D. (2021). The PRISMA 2020 statement: An updated guideline for reporting systematic reviews. *The BMJ*, *372*. 10.1136/bmj.n71 10.1136/bmj.n71 PMC8005924 33782057

[CR68] Palacio, F. X., Callaghan, C. T., Cardoso, P., Hudgins, E. J., Jarzyna, M. A., Ottaviani, G., Riva, F., Graco-Roza, C., Shirey, V., & Mammola, S. (2022). A protocol for reproducible functional diversity analyses. *Ecography,**2022*(11), Article e06287. 10.1111/ecog.06287

[CR69] Pascuale, D., Garello, N. A., Blettler, M. C., Rabuffetti, A. P., & Espinola, L. A. (2024). A bibliometric analysis of the invertebrates inhabiting the hyporheic zone: Too fragmented and biased knowledge? *Ecohydrology & Hydrobiology. Advance Online Publication.*10.1016/j.ecohyd.2024.05.002

[CR70] Patil, P. B., Maity, S., & Sarkar, A. (2022). Potential human health risk assessment of microplastic exposure: Current scenario and future perspectives. *Environmental Monitoring and Assessment,**194*(12), Article 898. 10.1007/s10661-022-10539-1 36251091 10.1007/s10661-022-10539-1

[CR71] Peralta-Maraver, I., Reiss, J., & Robertson, A. L. (2018). Interplay of hydrology, community ecology and pollutant attenuation in the hyporheic zone. *Science of the Total Environment,**610–611*, 267–275. 10.1016/j.scitotenv.2017.08.036 10.1016/j.scitotenv.2017.08.036 28803202

[CR72] Peter, K. T., Herzog, S., Tian, Z., Wu, C., McCray, J. E., Lynch, K., & Kolodziej, E. P. (2019). Evaluating emerging organic contaminant removal in an engineered hyporheic zone using high resolution mass spectrometry. *Water Research,**150*, 140–152. 10.1016/j.watres.2018.11.050 30508711 10.1016/j.watres.2018.11.050

[CR73] Pokhrel, S. R., Chhipi-Shrestha, G., Hewage, K., & Sadiq, R. (2022). Sustainable, resilient, and reliable urban water systems: Making the case for a “one water” approach. *Environmental Reviews,**30*(1), 10–29. 10.1139/er-2020-0090

[CR74] R Core Team. (2023). *R: A language and environment for statistical computing*. R Foundation for Statistical Computing. https://www.R-project.org/

[CR75] Redžović, Z., Erk, M., Gottstein, S., Perić, M. S., Dautović, J., Fiket, Ž, Brkić, A. L., & Cindrić, M. (2023). Metal bioaccumulation in stygophilous amphipod *Synurella ambulans* in the hyporheic zone: The influence of environmental factors. *Science of the Total Environment,**866*, Article 161350. 10.1016/j.scitotenv.2022.161350 36603643 10.1016/j.scitotenv.2022.161350

[CR76] Retter, A., Karwautz, C., & Griebler, C. (2021). Groundwater microbial communities in times of climate change. *Current Issues in Molecular Biology,**41*, 509–538. 33026361 10.21775/cimb.041.509

[CR77] Rinderhagen, M., Ritterhoff, J., & Zauke, G. P. (2000). Crustaceans as bioindicators. In *Biomonitoring of Polluted Water: Reviews on Actual Topics* (Environmental Research Forum, Vol. 9, pp. 161–194). Trans Tech Publications.

[CR78] Rivett, M. O., Ellis, P. A., Greswell, R. B., et al. (2008). Cost-effective mini drive-point piezometers and multilevel samplers for monitoring the hyporheic zone. *Quarterly Journal of Engineering Geology and Hydrogeology,**41*(1), 49–60. 10.1144/1470-9236/07-012

[CR79] Roveta, C., Annibaldi, A., Afghan, A., Calcinai, B., Di Camillo, C. G., Gregorin, C., Illuminati, S., Pulido Mantas, T., Truzzi, C., & Puce, S. (2021). Biomonitoring of heavy metals: The unexplored role of marine sessile taxa. *Applied Sciences,**11*(2), Article 580. 10.3390/app11020580

[CR80] Rutere, C., Knoop, K., Posselt, M., Ho, A., & Horn, M. A. (2020). Ibuprofen degradation and associated bacterial communities in hyporheic zone sediments. *Microorganisms,**8*(8), Article 1245. 10.3390/microorganisms8081245 32824323 10.3390/microorganisms8081245 PMC7464344

[CR81] Saavedra Cifuentes, E., Teitelbaum, Y., Arnon, S., Dallmann, J., Phillips, C. B., & Packman, A. I. (2023). Turbulence‐driven clogging of hyporheic zones by fine particle filtration. *Geophysical Research Letters,**50*(20), Article e2023GL105002. 10.1029/2023GL105002

[CR82] Sabale, R., Venkatesh, B., & Jose, M. (2023). Sustainable water resource management through conjunctive use of groundwater and surface water: A review. *Innovative Infrastructure Solutions,**8*(1), Article 17. 10.1007/s41062-022-00992-9

[CR83] Saccò, M., Mammola, S., Altermatt, F., Alther, R., Bolpagni, R., Brancelj, A., Brankovits, D., Fišer, C., Gerovasileiou, V., Griebler, C., Guareschi, S., Hose, G. C., Korbel, K., Lictevout, E., Malard, F., Martínez, A., Niemiller, M. L., Robertson, A., Tanalgo, K. C., & Reinecke, R. (2024). Groundwater is a hidden global keystone ecosystem. *Global Change Biology,**30*(1), Article e17066. 10.1111/gcb.17066 38273563 10.1111/gcb.17066

[CR84] Saeibehrouzi, A., Denissenko, P., Holtzman, R., Kantsler, V., & Abolfathi, S. (2025). Solute spreading enhancement by drainage-imbibition cycles in unsaturated porous media. *Water Research,**283*, Article 123741. 10.1016/j.watres.2025.123741 40381275 10.1016/j.watres.2025.123741

[CR85] Sagova-Mareckova, M., Boenigk, J., Bouchez, A., Cermakova, K., Chonova, T., Cordier, T., Eisendle, U., Elersek, T., Fazi, S., Fleituch, T., Frühe, L., Gajdosova, M., Graupner, N., Haegerbaeumer, A., Kelly, A.-M., Kopecky, J., Leese, F., Nõges, P., Orlic, S., & Stoeck, T. (2021). Expanding ecological assessment by integrating microorganisms into routine freshwater biomonitoring. *Water Research,**191*, Article 116767. 10.1016/j.watres.2020.116767 33418487 10.1016/j.watres.2020.116767

[CR86] Schälchli, U. (1992). The clogging of coarse gravel river beds by fine sediment. *Hydrobiologia,**235*, 189–197. 10.1007/BF00026211

[CR87] Schneider, A. S., Knüsel, M., & Altermatt, F. (2023). Assessment of occurrence, diversity, and biomass of macroinvertebrates in Swiss groundwater systems using citizen science data. *Subterranean Biology,**46*, 147–164. 10.3897/subtbiol.46.112569

[CR88] Shaikh, M., & Birajdar, F. (2024). Advancements in remote sensing and GIS for sustainable groundwater monitoring: Applications, challenges, and future directions. *International Journal of Research in Engineering Science and Management,**7*(3), 16–24.

[CR89] Sonne, A. T., Rasmussen, J. J., Höss, S., Traunspurger, W., Bjerg, P. L., & McKnight, U. S. (2018). Linking ecological health to co-occurring organic and inorganic chemical stressors in a groundwater-fed stream system. *Science of the Total Environment,**642*, 1153–1162. 10.1016/j.scitotenv.2018.06.119 30045497 10.1016/j.scitotenv.2018.06.119

[CR90] Staponites, L. R., Simon, O. P., Barták, V., & Bílý, M. (2022). Management effectiveness in a freshwater protected area: Long-term water quality response to catchment-scale land use changes. *Ecological Indicators,**144*, Article 109438. 10.1016/j.ecolind.2022.109438

[CR91] Stefanidis, K., & Papastergiadou, E. (2024). Ecological monitoring and assessment of freshwater ecosystems: New trends and future challenges. *Water,**16*(11), Article 1460. 10.3390/w16111460

[CR92] Steffen, W., Broadgate, W., Deutsch, L., Gaffney, O., & Ludwig, C. (2015). The trajectory of the Anthropocene: The great acceleration. *The Anthropocene Review,**2*(1), 81–98. 10.1177/2053019614564785

[CR93] Stoffers, T., Schultze, A. K., Ehlert, T., Kaiser, L., Scholz, M., & Nagelkerke, L. A. (2026). Challenges and opportunities in restoring European free-flowing rivers. *Nature Conservation,**62*, 355–381. 10.3897/natureconservation.62.173762

[CR94] Stride, B., Abolfathi, S., Bending, G. D., & Pearson, J. M. (2025). Hyporheic exchange processes of pore-scale microplastics. *Science of the Total Environment,**982*, Article 179573. 10.1016/j.scitotenv.2025.179573 40373686 10.1016/j.scitotenv.2025.179573

[CR95] Strona, G., Fattorini, S., Fiasca, B., Di Lorenzo, T., Di Cicco, M., Lorenzetti, W., Boccacci, F., & Galassi, D. M. (2019). Aqualife software: A new tool for a standardized ecological assessment of groundwater dependent ecosystems. *Water,**11*(12), Article 2574. 10.3390/w11122574

[CR96] Stubbington, R., Greenwood, A. M., Wood, P. J., Armitage, P. D., Gunn, J., & Robertson, A. L. (2009). The response of perennial and temporary headwater stream invertebrate communities to hydrological extremes. *Hydrobiologia,**630*, 299–312. 10.1007/s10750-009-9823-8

[CR97] Taglianti, A. V., Cottarelli, V., & Argano, R. (1969). Messa a punto di metodiche per la raccolta della fauna interstiziale e freatica. *Archivio Botanico e Biogeografico Italiano,**45*(14), 375–380.

[CR98] Thomle, J., Strickland, C., Johnson, T. C., Zhu, Y., & Stegen, J. (2020). A flux detection probe to quantify dynamic groundwater‐surface water exchange in the hyporheic zone. *Groundwater,**58*(6), 892–900. 10.1111/gwat.13001 10.1111/gwat.13001 32222074

[CR99] Tonina, D., & Buffington, J. M. (2023). Physical and biogeochemical processes of hyporheic exchange in alluvial rivers. In *Groundwater Ecology and Evolution* (pp. 61–87). Academic Press. 10.1016/B978-0-12-819119-4.15001-2

[CR100] Tonina, D., & Buffington, J. M. (2007). Hyporheic exchange in gravel bed rivers with pool‐riffle morphology: Laboratory experiments and three‐dimensional modeling. *Water Resources Research*. 10.1029/2005WR004328

[CR101] U.S. Environmental Protection Agency. (1997). *Guidance for future state ground water protection grants* [Memorandum from Robert Perciasepe to USEPA Regional Ground Water and Drinking Water Representatives].

[CR102] Van den Brink, P. J., Alexander, A. C., Desrosiers, M., Goedkoop, W., Goethals, P. L., Liess, M., & Dyer, S. D. (2011). Traits‐based approaches in bioassessment and ecological risk assessment: Strengths, weaknesses, opportunities and threats. *Integrated Environmental Assessment and Management,**7*(2), 198–208. 10.1002/ieam.109 20981837 10.1002/ieam.109

[CR103] Vörösmarty, C. J., McIntyre, P. B., Gessner, M. O., Dudgeon, D., Prusevich, A., Green, P., Glidden, S., Bunn, S. E., Sullivan, C. A., Reidy Liermann, C., & Davies, P. M. (2010). Global threats to human water security and river biodiversity. *Nature,**467*(7315), 555–561. 10.1038/nature09440 20882010 10.1038/nature09440

[CR104] Voulvoulis, N., Arpon, K. D., & Giakoumis, T. (2017). The EU Water Framework Directive: From great expectations to problems with implementation. *Science of the Total Environment,**575*, 358–366. 10.1016/j.scitotenv.2016.09.228 27744201 10.1016/j.scitotenv.2016.09.228

[CR105] Wang, Z., Zhang, Y., Kang, S., Yang, L., Shi, H., Tripathee, L., & Gao, T. (2021). Research progresses of microplastic pollution in freshwater systems. *Science of the Total Environment,**795*, Article 148888. 10.1016/j.scitotenv.2021.148888 34328911 10.1016/j.scitotenv.2021.148888

[CR106] Wang, L., Wang, Z., & Li, Y. (2022). Ecological restoration of hyporheic zone based on connectivity restoration: A review. *Advances in Water Science,**33*(6), 1009–1020. 10.14042/j.cnki.32.1309.2022.06.015

[CR107] Wang, H., Zhang, Z., Zheng, T., Chen, M., & Fang, Y. (2025). Recent trends in hyporheic zone research. *Environmental Earth Sciences,**84*(23), Article 701. 10.1007/s12665-025-12708-0

[CR108] Ward, A. S. (2016). The evolution and state of interdisciplinary hyporheic research. *Wires Water,**3*(1), 83–103. 10.1002/wat2.1120

[CR109] Ward, J. V., Voelz, N. J., & Harvey, J. H. (1989). *Groundwater faunas as indicators of groundwater quality: The South Platte River system* (No. 150). Colorado Water Resources Research Institute, Colorado State University.

[CR110] Water Act. (2007). *(Cth). Act No. 137 of 2007 as amended up to Act No. 5 of 2011, Com Law Authoritative Act C2011C00160*. Commonwealth of Australia. http://www.comlaw.gov.au

[CR111] Wilkinson, M. D., Dumontier, M., Aalbersberg, I. J. J., Appleton, G., Axton, M., Baak, A., Blomberg, N., Boiten, J.-W., Bonino da Silva Santos, L., Bourne, P. E., Bouwman, J., Brookes, A. J., Clark, T., Crosas, M., Dillo, I., Dumon, O., Edmunds, S., Evelo, C. T., Finkers, R., … Mons, B. (2016). The FAIR guiding principles for scientific data management and stewardship. *Scientific Data,**3*(1), 1–9. 10.1038/sdata.2016.18 10.1038/sdata.2016.18 PMC4792175 26978244

[CR112] Williams, D. D., Febria, C. M., & Wong, J. C. (2010). Ecotonal and other properties of the hyporheic zone. *Fundamental and Applied Limnology,**176*(4), Article 349.

[CR113] Winter, T. C. (2001). Ground water and surface water: The linkage tightens, but challenges remain. *Hydrological Processes,**15*(18), 3605–3606.

[CR114] Woessner, W. W. (2017). Hyporheic zones. In *Methods in Stream Ecology, Volume 1* (pp. 129–157). Academic Press.

[CR115] Wood, P. J., Gilvear, D. J., Willby, N., Robertson, A. L., Gledhill, T., & Boon, P. J. (2012). Improvements in understanding the contribution of hyporheic zones to biodiversity and ecological functioning of UK rivers. In *River Conservation and Management* (pp. 159–173). 10.1002/9781119961819.ch13

[CR116] Yan, Z., Zheng, X., Fan, J., Zhang, Y., Wang, S., Zhang, T., Sun, Q., & Huang, Y. (2020). China national water quality criteria for the protection of freshwater life: Ammonia. *Chemosphere,**251*, Article 126379. 10.1016/j.chemosphere.2020.126379 32171130 10.1016/j.chemosphere.2020.126379

[CR117] Yang, S., Xu, F., Wu, F., Wang, S., & Zheng, B. (2014). Development of PFOS and PFOA criteria for the protection of freshwater aquatic life in China. *Science of the Total Environment,**470*, 677–683. 10.1016/j.scitotenv.2013.09.094 24184545 10.1016/j.scitotenv.2013.09.094

[CR118] Zhou, Q., Chen, C., Zhang, G., Chen, H., Chen, D., Yan, Y., Shen, J., & Zhou, R. (2018). Real-time management of groundwater resources based on wireless sensor networks. *Journal of Sensor and Actuator Networks,**7*(1), Article 4. 10.3390/jsan7010004

