# Supplementary material for: Monitoring the hyporheic zone: a global review and strategic directions for improvement
Source: Environ Monit Assess. 2026 Apr 21;198(5):486. doi: 10.1007/s10661-026-15291-4 (PMC13099720; doi:10.1007/s10661-026-15291-4)
Supplement: Supplementary file 2 — (PDF 183 KB) [file 10661_2026_15291_MOESM2_ESM.pdf]

Monitoring the Hyporheic Zone: A Global Review and Strategic Directions for Improvement

Daniel da Silva Andrade[1*], Tiziana Di Lorenzo[3,4,5,6], Silvia Vendruscolo Milesi[7], Luiz Ubiratan Hepp[7], and Riccardo Mugnai [1,2,8]

[1] *Programa de Pós-Graduação em Ciências Ambientais, Universidade Federal do Maranhão (UFMA), BR 222 km 4, s/n, bairro Boa Vista, Chapadinha, MA, Brazil.*

*daniel.andhad@gmail.com; https://orcid.org/0000-0003-4608-090X**

[2] *Laboratório de Organismos Aquáticos, Curso Oceanografia, Centro de Ciências Biológicas e da Saúde (CCBS), Universidade Federal do Maranhão (UFMA), Av. dos Portugueses 1966, Cidade Universitária Dom Delgado, CEP 65080-805, São Luís, MA, Brazil.*

mugnai.riccardo@ufma.br; https://orcid.org/0000-0002-4476-3099

[3] *Research Institute on Terrestrial Ecosystems of the National Research Council of Italy (IRET CNR), 50019 Florence, Italy.*

tiziana.dilorenzo@cnr.it; https://orcid.org/0000-0002-3131-7049

[4] *National Biodiversity Future Center (NBFC), 90133 Palermo, Italy.*

[5] *"Emil Racovi¸tˇa" Institute of Speleology, 400535 Cluj-Napoca, Romania.*

[6] *Centre for Ecology, Evolution and Environmental Changes & CHANGE – Global Change and Sustainability Institute, and Departamento de Biologia Animal, Faculdade de Ciências, Universidade de Lisboa, Campo Grande, 1749-016 Lisbon, Portugal.*

[7] *Laboratório de Indicadores Ambientais, Universidade Federal de Mato Grosso do Sul (UFMS), Campus Três Lagoas, Três Lagoas, Brazil.*

silvia_milesi@yahoo.com.br; http://orcid.org/0000-0002-9568-6476

luizuhepp@gmail.com; http://orcid.org/0000-0002-8499-9549

[8]*Programa de Pós-Graduação em Biodiversidade e Conservação, Universidade Federal do Maranhão (UFMA), Av. dos Portugueses 1966, Cidade Universitária Dom Delgado, CEP 65080-805, São Luís, MA, Brazil.*

** Correspondence author:*

daniel.andhad@gmail.com; https://orcid.org/0000-0003-4608-090X

*Present address correspondence author:

-Laboratório de Plâncton, Departamento de Hidrobiologia, Universidade Federal de São Carlos. Rodovia Washington Luís, km 235, SP-310, 13565-905 São Carlos, SP, Brazil.

-Programa de Pós-graduação em Ciências Biológicas (Zoologia), Instituto de Biociências, Universidade Estadual Paulista "Júlio de Mesquita Filho" – UNESP, R. Prof. Dr. Antônio Celso Wagner Zanin, 250, Distrito de Rubião Junior, CEP 18618-689, Botucatu, SP, Brasil

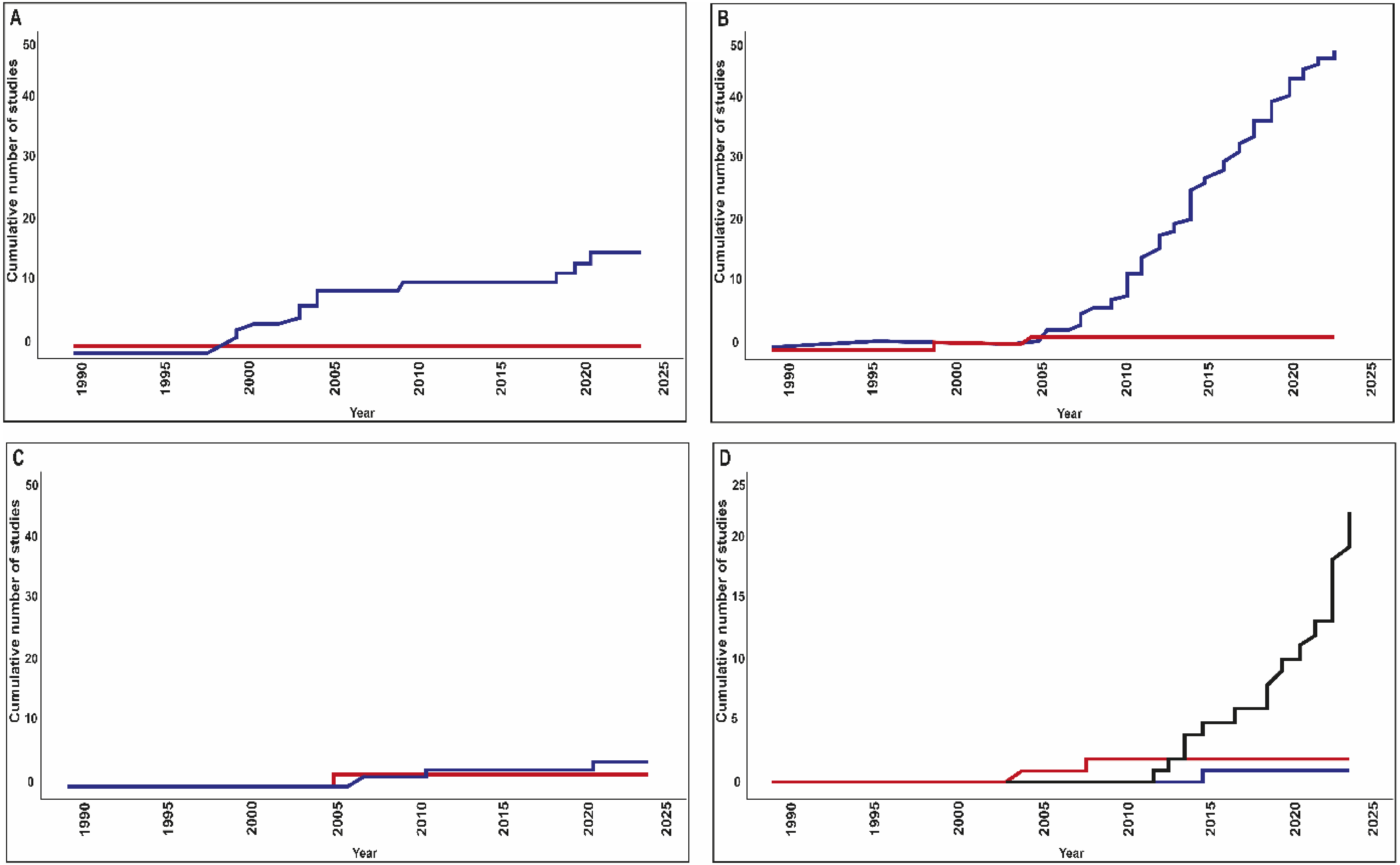
A
B
C
D
Cumulative number of studies
Year
0
10
20
30
40
50
5
15
25
1990
1995
2000
2005
2010
2015
2020
2025

Fig. S1. Cumulative number of studies per year in relation to the implementation of key environmental legislation. (A) North America (United States): studies before (red line) and after (blue line) the implementation of the 1996 legislation. (B) European Union: studies before (red line) and after (blue line) the implementation of the 2006 legislation. (C) Oceania (Australia): studies before (red line) and after (blue line) the implementation of the 2007 legislation. (D) No legislation areas: Asia (black line), Central and South America (blue line), and Africa (red line).
